# SARS-CoV-2 infection engenders heterogeneous ribonucleoprotein interactions to impede translation elongation in the lungs

**DOI:** 10.1038/s12276-023-01110-0

**Published:** 2023-11-01

**Authors:** Junsoo Kim, Daehwa Youn, Seunghoon Choi, Youn Woo Lee, Dulguun Sumberzul, Jeongeun Yoon, Hanju Lee, Jong Woo Bae, Hyuna Noh, Dain On, Seung-Min Hong, Se-Hee An, Hui Jeong Jang, Seo Yeon Kim, Young Been Kim, Ji-Yeon Hwang, Hyo-Jung Lee, Hong Bin Kim, Jun Won Park, Jun-Won Yun, Jeon-Soo Shin, Jun-Young Seo, Ki Taek Nam, Kang-Seuk Choi, Ho-Young Lee, Hyeshik Chang, Je Kyung Seong, Jun Cho

**Affiliations:** 1 Center for RNA Research, Institute for Basic Science (IBS), Seoul National University, Seoul, Republic of Korea; 2 Interdisciplinary Program in Bioinformatics, Seoul National University, Seoul, Republic of Korea; 3 Department of Biomedical Science and Engineering, Gwangju Institute of Science and Technology (GIST), Gwangju, Republic of Korea; 4 Korea Mouse Phenotyping Center (KMPC), Seoul National University, Seoul, Republic of Korea; 5 Laboratory of Developmental Biology and Genomics, Research Institute for Veterinary Science, and BK 21 PLUS Program for Creative Veterinary Science Research, College of Veterinary Medicine, Seoul National University, Seoul, Republic of Korea; 6 Department of Nuclear Medicine, Seoul National University Bundang Hospital, Seongnam, Republic of Korea; 7 School of Biological Sciences, Seoul National University, Seoul, Republic of Korea; 8 Laboratory of Avian Diseases, Research Institute for Veterinary Science, and BK 21 PLUS Program for Creative Veterinary Science Research, College of Veterinary Medicine, Seoul National University, Seoul, Republic of Korea; 9 Preclinical Research Center, Seoul National University Bundang Hospital, Seongnam, Republic of Korea; 10 Department of Periodontology, Section of Dentistry, Seoul National University Bundang Hospital, Seongnam, Republic of Korea; 11 Department of Internal Medicine, Seoul National University Bundang Hospital, Seoul National University College of Medicine, Seongnam, Republic of Korea; 12 Division of Biomedical Convergence, College of Biomedical Science, Kangwon National University, ChunCheon, Republic of Korea; 13 Laboratory of Veterinary Toxicology, College of Veterinary Medicine, Seoul National University, Seoul, Republic of Korea; 14 Severance Biomedical Science Institute, Graduate School of Medical Science, Brain Korea 21 Project, Yonsei University College of Medicine, Seoul, Republic of Korea; 15 Department of Nuclear Medicine, Seoul National University, College of Medicine, Seoul, Republic of Korea; 16 Interdisciplinary Program and BIO MAX Institute, Seoul National University, Seoul, Republic of Korea

**Keywords:** Ribosome, Viral infection

## Abstract

Translational regulation in tissue environments during in vivo viral pathogenesis has rarely been studied due to the lack of translatomes from virus-infected tissues, although a series of translatome studies using in vitro cultured cells with viral infection have been reported. In this study, we exploited tissue-optimized ribosome profiling (Ribo-seq) and severe-COVID-19 model mice to establish the first temporal translation profiles of virus and host genes in the lungs during SARS-CoV-2 pathogenesis. Our datasets revealed not only previously unknown targets of translation regulation in infected tissues but also hitherto unreported molecular signatures that contribute to tissue pathology after SARS-CoV-2 infection. Specifically, we observed gradual increases in pseudoribosomal ribonucleoprotein (RNP) interactions that partially overlapped the trails of ribosomes, being likely involved in impeding translation elongation. Contemporaneously developed ribosome heterogeneity with predominantly dysregulated 5 S rRNP association supported the malfunction of elongating ribosomes. Analyses of canonical Ribo-seq reads (ribosome footprints) highlighted two obstructive characteristics to host gene expression: ribosome stalling on codons within transmembrane domain-coding regions and compromised translation of immunity- and metabolism-related genes with upregulated transcription. Our findings collectively demonstrate that the abrogation of translation integrity may be one of the most critical factors contributing to pathogenesis after SARS-CoV-2 infection of tissues.

## SARS-CoV-2 infection: disruption of translation integrity

This study reveals that SARS-CoV-2 exhibits distinct profiles of viral gene expression in tissue microenvironments, with lower levels of subgenomic RNAs and subdued expansion compared to cell lines. Researchers discovered pseudoribosomal ribonucleoprotein (RNP) interactions developing in the lungs during SARS-CoV-2 pathogenesis, which may contribute to the accumulation of small Ribo-seq reads and affect translation elongation. Ribosome heterogeneity with compromised 5 S rRNP association was also observed, suggesting a potential strategy for coping with SARS-CoV-2 pathogenesis by resolving RBP complexes. The findings provide valuable insights into the molecular mechanisms underlying SARS-CoV-2 infection and potential therapeutic targets for COVID-19 treatment.

## Introduction

Coronavirus disease 2019 (COVID-19), caused by severe acute respiratory syndrome coronavirus 2 (SARS-CoV-2)^1,2^, has caused an unprecedented pandemic with >760 million infections and more than 6.8 million deaths to date. Despite the enhanced protection provided by SARS-CoV-2 vaccines, the infection has not been fully constrained, and options to treat the disease are still limited. The development of a proven therapy to treat people postinfection is critical, necessitating a fundamental understanding of viral behaviors and host responses during pathogenesis after virus invasion.

To explain the gene regulation underlying the pathogenesis of SARS-CoV-2 infection, a number of transcriptome studies on SARS-CoV-2-infected animals and COVID-19 patients have been performed^3–7^. However, a pitfall of transcriptome-based approaches is that mRNA level changes do not always correlate with protein level changes^8,9^. Given that several viruses are known to disrupt host protein synthesis^10–13^ and that antiviral responses involve gene regulation, such as the regulation of mTOR signaling, are mediated in a translation-dependent manner^14^, scrutinizing gene translation is essential to understanding virus‒host interactions at the molecular level.

Ribosome profiling (Ribo-seq) is a deep sequencing-based technique that captures in vivo interactions between ribosomes and RNAs as ribosome-protected fragments (RPFs) after RNase digestion^15^. By virtue of the precise determination of ribosome positions across transcripts and quantitative estimation of translation activity per gene on a genome-wide scale, ‘translatomes’ constructed by Ribo-Seq have elucidated hidden features of gene translation in diverse biological contexts^16–20^. In particular, this powerful technique has been exploited to identify viral ORFs and explore the regulation of viral gene translation as well as that of host genes upon the infection with various viruses, such as cytomegalovirus, herpes simplex virus, influenza virus, and murine coronavirus^21–24^.

In 2021, Finkel et al. applied Ribo-seq to SARS-CoV-2-infected cell lines (the Vero E6 and Calu-3 cell lines), thereby evaluating the protein-coding capacities of canonical and noncanonical ORFs in viral transcripts^25^. They subsequently measured viral and host mRNA translational activity in the early postinfection period (from 0 to 8 h post infection), discovering that impaired nuclear export and accelerated degradation of host mRNAs are likely the strategies mediated by SARS-CoV-2 virus to impede host protein synthesis^26^. Kim et al. performed translatome studies with infected cell lines over an extended time period (0–36 h), identifying secondary initiation sites of SARS-CoV-2 transcript translation^27^. More recently, two independent translatome studies of in vitro cultured cells with a lung epithelium origin reported that several cytokines and innate immune factors were translated at a low rate in SARS-CoV-2-infected cells^28,29^.

These previous translatome studies improved our understanding of mechanisms by which viral and host gene translation affects viral pathogenesis; however, the studies were performed with in vitro experiments involving virus interactions with a single type of host cell. In in vivo environments of virus-infected tissues, diverse types of cells, such as immune cells, communicate with infected cells; these cells influence each other, which may lead to distinct viral and host gene regulatory trajectories that cannot be properly assessed when in vitro systems are used^30^. Moreover, prominent pathological features observed in human patients with severe COVID-19 symptoms and in animal models of SARS-CoV-2-induced pathology include imbalances in the immune response and tissue homeostasis that cannot be fully recapitulated in cell line models of infection, underscoring that viral and host translation within the tissue microenvironment needs to be evaluated to comprehend SARS-CoV-2 pathophysiology. However, among all the published translatome studies on viral infection, including SARS-CoV-2 infection, none currently covers translational regulation in vivo.

We therefore applied tissue-optimized Ribo-seq to the lungs of severe-COVID-19 model mice to establish the first temporal profiles of gene translation in vivo during SARS-CoV-2 pathogenesis. Our data reveal that the translation of viral and host genes in the tissue environment is markedly different from that observed with in vitro systems. In addition to identifying the distinct targets of translation modulation in vivo in infected tissues, we discovered previously unknown molecular signatures that represent the tissue pathology of SARS-CoV-2 infection: pseudoribosomal ribonucleoprotein (RNP) complexes, ribosome heterogeneity with deviated 5 S rRNP association, and impediment of translation elongation.

## Materials and Methods

### Mice used for studying SARS-CoV-2 infection

The mice used in this study (8 weeks old, male) were obtained from the Jackson Laboratory (B6.Cg-Tg(K18-ACE2)2Prlman/J). All protocols were approved by the Institutional Animal Care and Use Committee of the Seoul National University Bundang Hospital (IACUC number BA-2008-301-071-05). The Ji Seok Young Research Center is fully accredited by the Association for Assessment and Accreditation of Laboratory Animal Care. All animals were cared for in accordance with the Institute for Laboratory Animal Research Guide for the Care and Use of Laboratory Animals, eighth edition. The Seoul National University Bundang Hospital Institutional Biosafety Committee approved the procedures for sample handling, inactivation, and transfer from animal biosafety Level 3 (ABSL3) containment. The mice were lightly anesthetized with ketamine (20 mg/kg) and xylazine (10 mg/kg) during the infection procedure. All mice were infected intranasally with virus in a total volume of 50 μl of DMEM. The weight and temperature of the mice were monitored daily. The mice were sacrificed in a CO_2_ chamber on Days 0, 1, 2, 5, and 7 post infection (dpi). All animal experiments involving SARS-CoV-2 were performed in an ABSL3 laboratory at the Seoul National University Bundang Hospital.

### SARS-CoV-2 preparation and titration

The original Wuhan (WA1) strain of SARS-CoV-2 (accession number: NCCP43326/Korea) was procured from the Korea Centers for Disease Control and Prevention (KDCDC03/2020), and Vero E6 cells (CRL-1586) were procured from the Korea Microbial Resource Center (KCTC). Vero E6 cells were maintained in Dulbecco’s modified Eagle’s medium (DMEM) (Life Technologies, CA, USA), which contained 10% fetal bovine serum (FBS) (Life Technologies). On the third day, the Vero E6 cells were inoculated with SARS-CoV-2 to test the cytopathic effect of the strain.

The virus titer was measured by plaque assay. Vero E6 cells were seeded in 12-well plates at a concentration of 3 × 10^5^ cells per well and incubated. A monolayer formed one day prior to the plaque assay. The cells were infected for 1 h in duplicate with 10-fold serial dilutions of SARS-CoV-2 and overlaid with 0.3% SeaPlaque (LONZA, Basel, Switzerland) agarose medium containing 2% FBS. After 72 h of incubation, the cells infected with virus were fixed with 4% paraformaldehyde for 1 h and then stained with a crystal violet solution (Sigma–Aldrich, 548-62-9). The infectious virus titers were measured, and the data are reported in plaque-forming units (PFU) per ml.

### Construction of Ribo-seq libraries from SARS-CoV-2-infected lungs

The lung tissues isolated from sacrificed mice were homogenized within lysis buffer (10 mM/mL Tris-HCl, pH 7.4 (AM9850G, AM9855G Invitrogen); 5 mM/mL MgCl_2_ (#AM9530G Ambion); 100 mM/mL KCl (#AM9640G Ambion); 2 mM/mL dithiothreitol (DTT, #707265 ML Thermo Fisher); 300 μg/mL cycloheximide (CHX, #C1988-1G Sigma‒Aldrich); 1% Triton X-100 (#T8787 Sigma‒Aldrich); 1X protease inhibitor (#P3100-001 GenDEPOT); 2 μL/mL SUPERase inhibitor (#AM2696, Invitrogen); and 2 μL/mL RNase inhibitor (#AM2694 Invitrogen)). The treated cells were incubated for 5 min on ice followed by incubation at 4 °C for 30 min with additional lysis buffer containing CHX at a concentration that was threefold less than the original concentration. After incubation, the samples were centrifuged, and the supernatant was divided into two parts. One-half of the supernatant was treated with TRIzol LS (#10296028 Invitrogen) to isolate RNA to be used to generate a transcriptome library (RNA-Seq) using TruSeq Stranded Total RNA Library Prep Gold (#20020599 Illumina), and one-half was treated with 0.1 U/mL RNase I (#EN0601 Thermo Fisher) for 30 min at 25 °C to digest mRNA regions that were not associated with ribosomes. To remove the digested mRNA fragments and collect the ribosome-protected mRNA-containing lysate, samples were passed through illustra^TM^ MicroSpin S400 HR Columns (#27-5140-01 Cytiva), which had been prewashed with a polysome buffer (20 mM HEPES KOH (#BP299100 Fisher BioReagents), pH 7.4; 5 mM MgCl2; 100 mM KCl; 2 mM DTT; and 100 μg/mL CHX). Then, they were treated with TRIzol LS. Ribosomal RNAs were depleted using a Ribo-Zero Gold Kit with TruSeq Stranded Total RNA Library Prep Gold (#20020599 Illumina) according to the manufacturer’s protocol, and they were precipitated in 100% ethanol (#100983 Supelco) at -80 °C. Samples were labeled with the radioisotope γ-P^32^ ATP (#NEG-502H-1 PerkinElmer) via the action of the T4 Polynucleotide kinase (T4 PNK, #M0201L New England Biolabs) and separated on a 10% denaturing Urea-PAGE (#U5128 Sigma‒Aldrich) gel. The signals were captured on Fujifilm imaging plates (#28956478 GE Healthcare). The radioisotope signals were visualized with an Amersham Typhoon Biomolecular imager (v2.0.0.6 GE Healthcare) and analyzed using MultiGauge (v3.0) software. After protein separation, samples of approximately 30 nt were excised from the gel, and the RNA was purified. The purified samples were then dephosphorylated with Antarctic Phosphatase (#M0289S New England Biolabs) and biochemically relabeled with γ-P^32^ ATP using the T4 PNK reaction, which attaches the phosphate to the free hydroxyl 5′ end of the RNA. Notably, 10 mM ATP (#P0756S New England Biolabs) was added to compensate for the lack of γ-P^32^ ATP. The bound labeled samples were separated from the free ATP in a 10% denaturing urea-PAGE gel followed by RNA excision and purification, as described above. Adapter ligation and PCR amplification were performed using a SomaGenics RealSeq®-AC miRNA Library Kit (#500-00048 RealSeq Biosciences) according to the manufacturer’s protocol. The desired adapter-ligated PCR products were purified with SPRIselect magnetic beads as per the manufacturer’s protocol, left side selection. Libraries were sequenced by the NovaSeq 6000 system.

### Computational analyses and other experiments

Details of the computational analyses and other experimental procedures are described in the supplemental material.

## Results

### Distinct behaviors of SARS-CoV-2 were identified via tissue translatome and transcriptome profile analyses

**Fig. 1:**
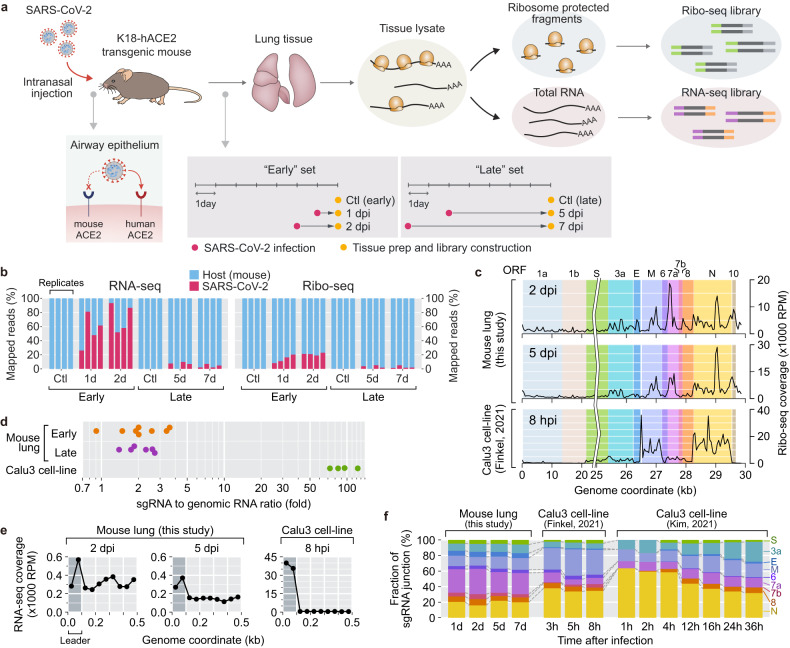
Transcriptome and translatome landscapes of SARS-CoV-2 in tissue microenvironments. **a** Experimental workflow. Ribo-seq and RNA-seq libraries were prepared using lung tissue lysates from K18-hACE2 transgenic mice infected with SARS-CoV-2. **b** The percentages of the RNA-seq and Ribo-seq reads that were mapped to the viral (red) or host (blue) genomes. Each bar represents an individual library of one replicate per condition. **c** Density maps of the Ribo-seq reads across the SARS-CoV-2 genome. **d** Ratios of sgRNAs to gRNA estimated by the proportions of TRS-L spanning reads in the RNA-seq datasets of mouse tissues and human cell lines. **e** Read coverages of the mouse tissue and human cell line RNA-seq libraries around the 5′ end of the SARS-CoV-2 genome. The 5′ leader sequence is dark gray. **f** Temporal profiles of individual SARS-CoV-2 sgRNA levels in mouse tissues and human cell lines.

To investigate the translation and transcription of viral and host genes in the tissues infected with SARS-CoV-2, we applied Ribo-seq in parallel with RNA-seq to lungs of K18-hACE2 transgenic mice after intranasal inoculation with virus (1 × 10^5^ PFU of the original Wuhan SARS-CoV-2 strain; Fig. 1a). The model mouse line expresses human angiotensin I-converting enzyme 2 (hACE2) in epithelial lineages including airway epithelium under the control of the cytokeratin-18 promoter (K18), and the pathological signs of severe COVID-19, such as pneumonia and pulmonary dysfunction, has been reported to be recapitulated in these mice in several studies^5,31,32^. After SARS-CoV-2 infection, the mice presented with marked decreases in weight loss and body temperature, becoming moribund at approximately 5–7 dpi (Supplementary Fig. 1a, b). The infected mice developed pulmonary inflammation that was characterized by the accumulation of mononuclear cells and neutrophils predominantly in perivascular areas at 1–4 dpi, and the inflammation expanded into alveolar spaces as perivascular and interstitial edema developed at 5–7 dpi (Supplementary Fig. 1c, d). The pathological phenotypes of 1–2 dpi and 5–7 dpi were noticeably different from each other, implying that the virus‒host interactions were markedly different. We thus prepared Ribo-seq and RNA-seq libraries 1 and 2 dpi, representing the early phase of SARS-CoV-2 pathogenesis (the early dataset), and 5 and 7dpi, representing the late phase of SARS-CoV-2 pathogenesis (the late dataset) (Fig. 1a).

To examine the dynamics of SARS-CoV-2 infection expansion and viral activity within the tissue microenvironments, we estimated the proportions of the Ribo-seq and RNA-seq reads that aligned to the mouse and SARS-CoV-2 genomes (Fig. 1b). The frequencies of viral genome-mapped reads in both the Ribo-seq and RNA-seq libraries representing early phase infection showed an increasing trend, indicating that the number of SARS-CoV-2 virions was continuously expanding during the early pathogenesis. The percentage of the Ribo-seq reads aligned to the viral genome (5–25%) was much lower than that of the RNA-seq read counterparts (30–85%), implying that the majority of the viral transcripts were probably not being translated by ribosomes. In the late Ribo-seq and RNA-seq library datasets, a minor portion of the reads (fewer than 10%) were aligned to the viral genome, which were very distinct from the observations in the datasets of the in vitro cultured cells, in which the proportion of SARS-CoV-2 virus-infected cells was continuously expanding with no meaningful abatement^25–27^. These results demonstrated that the proliferation or survival of SARS-CoV-2 virus in the mouse tissue was effectively restrained at some point before cells entered the late phase, in contrast to the pattern observed in cell lines.

The density maps of the Ribo-seq reads across the SARS-CoV-2 genome (Fig. 1c and Supplementary Fig. 1g) revealed that the tissue Ribo-seq libraries included higher read densities in the ORF1a and b regions and lower densities in the sgRNA region compared to the cell line counterparts, which alludes to relatively less expression of sgRNAs in the tissue microenvironment. In line with these observations, the ratios of sgRNA to gRNA (Fig. 1d) and the extent of the read stacks on leader sequence regions, representing the number of sgRNA junctions (Fig. 1e), were considerably lower in the tissue RNA-seq libraries than in the cell line datasets, indicating lower levels of steady-state SARS-CoV-2 sgRNAs. The lower ratio of sgRNA to gRNA may be attributable to restricted production of sgRNAs within infected hosts or active engulfment and degradation of SARS-CoV-2 gRNAs by the accumulating immune cells. Either potential mechanism may contribute to the suppressed expansion of SARS-CoV-2 in the late phase dataset representing 5 and 7 dpi (Fig. 1b).

Principal component analysis (PCA) of the viral translatome and transcriptome profiles (Supplementary Fig. 1e, f) demonstrated that individual viral genes were differentially expressed in tissues and cell lines. We estimated the proportions of individual sgRNA junctions, which represent the relative abundances of corresponding sgRNAs. While the cell line transcriptomes showed a predominance of the sgRNAs M, N, and ORF3a, the tissue transcriptomes showed relatively lower proportions of these sgRNAs and moderate predominance of 7a (Fig. 1f). Taken together, the data show that SARS-CoV-2 virus exhibited distinct viral gene expression in the tissue microenvironment, which may either result in or be a result of tissue-specific virus‒host interactions.

### Pseudoribosomal ribonucleoprotein interactions develop during SARS-CoV-2 pathogenesis

**Fig. 2:**
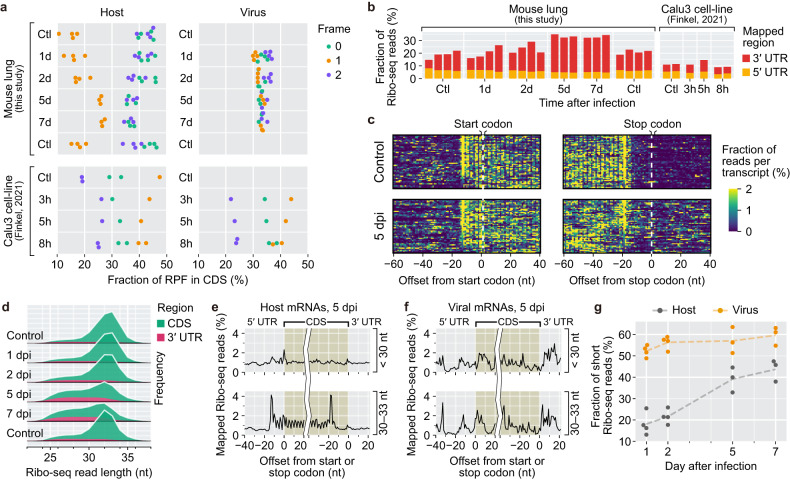
Pseudoribosomal RNP interactions manifest in the lungs during SARS-CoV-2 pathogenesis. **a** Frame distributions of the 5′ ends of Ribo-seq reads within codons of all nonmitochondrial mRNAs. **a**, **b**, **g**, one replicate in the 5 dpi group and the other replicate in the 7 dpi group were excluded due to significant library quality issues. For a more detailed explanation, please refer to the supplementary methods. **b** The percentage of Ribo-seq reads aligned to the 3′ UTR and 5′ UTR in each sample. **c** Density maps of the Ribo-seq reads across the start codons and the stop codons of abundant mRNA transcripts. The alignment coordinates were determined on the basis of the 5′ ends of individual Ribo-seq reads. The number of each coordinate was normalized by the total count per gene. **d** Length distributions of the Ribo-seq reads that were uniquely aligned to CDSs and 3′ UTRs. **e**, **f** Metagene analysis of the long (30–33 nt) and short (<30 nt) Ribo-seq reads around start codons and stop codons of host nuclear mRNAs (**e**) and SARS-CoV-2 transcripts (**f**). **g** Relative fractions of the short Ribo-seq reads uniquely mapped to CDSs of host nuclear mRNAs and SARS-CoV-2 transcripts.

We noted a number of Ribo-seq read peaks at promiscuous positions across the SARS-CoV-2 genome that had not been detected in the cell line translatome assay (Fig. 1c and Supplementary Fig. 1g). The majority of Ribo-seq reads were thought to be from RNA fragments protected by their associated ribosomes (RPFs), but through sucrose sedimentation or gel filtration used in the Ribo-seq protocols, other ribonucleoprotein (RNP) complexes with biochemical properties similar to those of canonical ribosomes can be isolated. We, therefore, examined frame periodicities (i.e., three nucleotide periodicities) and CDS (coding sequence) enrichment in the Ribo-seq reads to assess the contributions of authentic RPFs to the tissue Ribo-seq libraries (Fig. 2a, b). Surprisingly, the frame periodicities of the infected tissue were considerably compromised compared to those of the uninfected control tissues. The periodicity losses in host genes were more prominent in the late phase, indicating that the interactions between pseudoribosomal RNPs and host RNAs developed progressively during SARS-CoV-2 pathogenesis. For viral genes, the periodicities were almost completely abolished at all time points (Fig. 2a) despite varied frame usages of individual sgRNAs (Supplementary Fig. 2a). In addition, and more surprisingly, we observed that significantly higher proportions of the Ribo-seq reads from the SARS-CoV-2-infected tissues than from the uninfected controls were aligned to UTRs in host genes, showing an increasing trend postinfection (Fig. 2b, c). These results indicate that the tissue Ribo-seq libraries included a portion of RNAs that were unlikely to be associated with or translated by conventional ribosomes as SARS-CoV-2 pathogenesis progressed.

To dissect the biochemical properties of the pseudoribosomal RNPs that were associated with the attenuated frame periodicities and UTR enrichment in host transcripts, we analyzed the lengths of the Ribo-seq reads that were aligned to CDSs and UTRs. Intriguingly, the analysis revealed varied degrees of protection from RNase digestion for the CDS- and UTR-mapped reads (Fig. 2d). Although the uninfected controls harbored a dominant population of CDS mapped reads with a narrow range of longer sequences (~32 nt on average), the infected tissue dataset revealed additional populations with a broad range of shorter length sequences that were mapped to UTRs as well as CDSs. We divided the Ribo-seq reads into two groups based on read length, namely, into long (30–33 nt) and short (<30 nt) read groups, and performed metagene alignment across start and stop codons with the reads of each group. In contrast to the long Ribo-seq reads following the putative patterns of conventional RPFs, the short Ribo-seq reads were broadly distributed across 5′ UTRs, CDSs, and 3′ UTRs without periodicity (Fig. 2e), suggesting that the anomalous features of the infected tissue Ribo-seq libraries are likely attributable to the accumulation of the short reads. This speculation Is supported by the considerable positive correlations among Frame 1 usage, 3′ UTR enrichment, and short read length proportion of individual genes (Supplementary Fig. 2c). The temporal kinetics of the proportion of short read revealed that the number of these sequences progressively increased throughout the pathogenic period; similarly, the number of the two other abnormal features were increased (Fig. 2g). These results collectively demonstrate that the noncanonical Ribo-seq reads for host genes originate from and represent interactions between corresponding host transcripts and ribosome-like RNP complexes that are formed during pathogenesis.

Viral genes harbored much higher proportions (>50%) of short Ribo-seq reads than host genes in all the time point datasets (Fig. 2g), and both the long and short reads failed to recapitulate the conventional movements of ribosomes (Fig. 2f). These findings imply that, even on the first day after infection, the majority of viral Ribo-seq reads did not originate from canonical ribosome–RNA associations. The aforementioned periodicity losses for viral genes (Fig. 2a) and the lack of a preferential frame in ORF1ab (Supplementary Fig. 2d) also support this speculation. Noncanonical Ribo-seq read peaks were more highly distributed across viral transcripts than host UTRs (Supplementary Fig. 2b), implying that the aberrant RNP interactions may preferentially and frequently occur across viral transcripts or that the origins of these interactions, namely, pseudoribosomal RNPs, for viral and host genes may be different.

The Ribo-seq datasets of the SARS-CoV-2-infected cell lines did not show noticeable accumulation of short reads (data not shown) or UTR enrichment (Fig. 2b and Supplementary Fig. 2e) after infection, and they showed moderately compromised periodicities in both host and viral transcripts (Fig. 2a). These discrepancies between the tissue and cell line datasets suggest that the development of the pseudoribosomal RNP interactions largely took place in lung tissues, rather than in cell lines, after SARS-CoV-2 infection.

### The pseudoribosomal RNP-associated genes exhibit impeded translational elongation in infected tissues

**Fig. 3:**
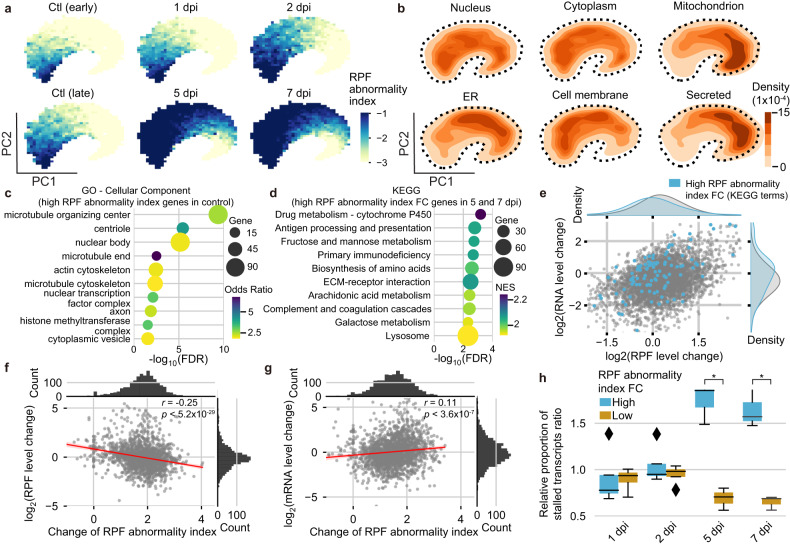
Pseudoribosomal RNP interactions are associated with translational suppression of transcriptionally activated genes. **a**, **b** t-SNE visualization of host nuclear genes (*n* = 9255) embedded in a 2D plot based on RAIs of all the individual Ribo-seq libraries. Each coordinate is colored according to **a** the RAI at each time point and **b** protein localization annotated in the UniProt database. **c** Gene Ontology (GO) term enrichment analysis using EnrichR for the top 20% of genes with the highest RAIs in uninfected controls. The top 10 GO terms of cellular compartments that were significantly enriched (adjusted *P* value < 0.05) are presented. The color gradients and circle sizes represent the odds ratios and numbers of overlapping genes, respectively. **d** Kyoto Encyclopedia of Genes and Genomes (KEGG) pathway enrichment analysis based on the RAIs of the late phase samples (5 and 7 dpi) versus the respective controls. The top 10 significantly enriched terms with an FDR below 0.05 are displayed. The color gradients and circle sizes represent normalized enrichment scores (NESs) and the numbers of overlapping genes, respectively. **e** A scatter plot representing the changes in RPF (x-axis) versus RNA (y-axis) levels in the samples taken 5 and 7 dpi compared to the uninfected controls. The sky blue dots represent the genes associated with overrepresented KEGG terms, and the gray dots represent all other genes. **f**, **g** Scatter plots representing the changes in RAI (*x* axis) versus the changes in RPF (**f**) and mRNA (**g**) levels (*y* axis) of samples taken 5 and 7 dpi compared to uninfected controls. Each dot represents an individual gene. Linear regression analysis was performed to assess the correlation and significance between RPF level changes and RAI. **h** Box plot showing the relative proportions of stalled transcripts at each time point for the genes with high (top 25%) and low (bottom 25%) RAI fold changes (FCs). The rhombuses indicate the outliers of all replicates per condition. Mann‒Whitney *U* test *P* values are denoted by asterisks. Significant differences are indicated by [*] *P* < 0.05.

To assess the target specificities of the pseudoribosomal RNP interactions, we quantified the putative proportions of the noncanonical Ribo-seq reads for individual genes based on the extent of short read ratio, Frame 1 usage, and 3′ UTR enrichment (RPF abnormality indices, RAIs) (Supplementary Table 1). Visualizing the temporal profiles of the RAIs with t-distributed stochastic neighbor embedding (t-SNE) revealed that the abnormalities progressively developed and spread across whole genes throughout SARS-CoV-2 pathogenesis (Fig. 3a). Notably, a portion of the genes were found at basally high proportions in the noncanonical Ribo-seq reads, including those in the uninfected controls, which was validated by pairwise comparisons between each time point and the respective control (Supplementary Fig. 3a). These observations suggest at least two possible origins for the noncanonical Ribo-seq reads that contribute to the initially high level of RAIs that progressively accrue. By overlaying the areas of subcellular localization (Fig. 3b), no pattern was found for any organelle.

The genes initially harboring high levels of RAIs were markedly overrepresented in microtubule-related Gene Ontology (GO) terms, suggesting that the origin of the basal noncanonical Ribo-seq reads might be associated with microtubule-related cellular functions (Fig. 3c). Intriguingly, these genes exhibited higher sensitivities to nonsense-mediated decay (NMD, Supplementary Fig. 3e) and showed high intron retention ratios (Supplementary Fig. 3d). In specific biological contexts, certain microtubule complexes accompanying RNA bindings are involved in transports of microtubule constituent-coding transcripts for their local translation or of aberrantly spliced mRNAs with retained introns to p-bodies where NMD takes place. The basal noncanonical Ribo-seq reads may conceivably be attributable to the molecules that interact with RNPs that are associated with the microtubule complexes.

The infection-triggered elevation of RPF abnormalities was observed broadly across most genes, but the quantitative assessment of RAIs for individual genes indicated they acquired the aberrant RNP interactions to different magnitudes (Fig. 3a and Supplementary Fig. 3a). We performed gene set enrichment analysis (GSEA)^33^ to identify for biological properties that are associated with biased increases in RPF abnormalities. The GSEA results revealed overrepresented multiple immune response- and metabolism-associated terms, such as “antigen processing and presentation” and “fructose and mannose metabolism” (Fig. 3d and Supplementary Fig. 3f). Notably, the RNA levels of the genes exhibited an overall increase in RNA levels, but to a lesser extent or decrease in RPF levels in the late phase (Fig. 3e), which led us to hypothesize that the aberrant RNP interactions are probably involved in the translational suppression of transcriptionally activated genes. In line with this hypothesis, the genes with high RAI increases exhibited decreasing and increasing trends in RPF (Fig. 3f and Supplementary Fig. 3g) and RNA levels (Fig. 3g and Supplementary Fig. 3h), respectively, over the time course (Supplementary Table 2). Given that the pseudoribosomal RNP interactions took place in the CDSs as well as UTRs, we examined whether the RNP–mRNA binding interferes with ribosome movement along a CDS during translation elongation. Notably, we observed that more potent ribosome stalling developed for the gene with high RAI increases as the pathogenesis proceeded (Fig. 3h), supporting the idea that translation is repressed by pseudoribosomal RNPs and that these molecules are conceivably involved in hindering the activities of elongating ribosomes.

To dissect sequence determinants of RPF abnormalities, we examined the association of the RAI with different properties of mRNA sequences, including their length, CG abundance, and purine ratio in CDS and UTRs. The length and CG ratio of a CDS were positively correlated with the RAI of the corresponding genes in the uninfected control group, alluding to the reliability of these properties as contributors to the basal RAI (Supplementary Fig. 3b). For the accruing RAIs, we observed strong negative correlations between RAI increases in the late phase and the CDS length and 5′ UTR CG proportion of individual genes (Supplementary Fig. 3c). These results suggest that pseudoribosomal RNP complexes may prefer transcripts with short ORFs and high AU frequencies in the 5′ UTR.

### Ribosome heterogeneity with compromised 5 S rRNP association manifests during SARS-CoV-2 pathogenesis

**Fig. 4:**
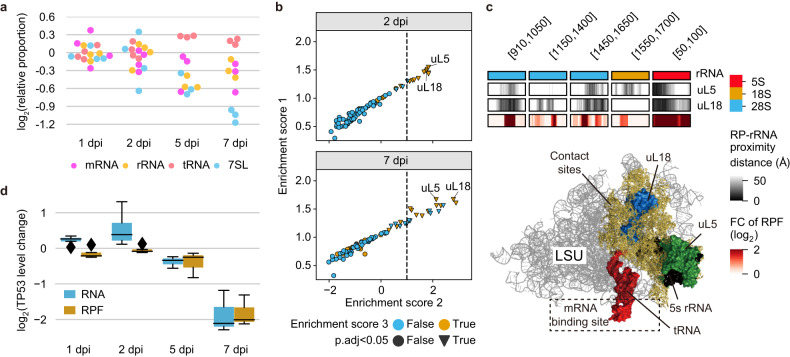
Ribosome heterogeneity with dysregulated rRNP associations in SARS-CoV-2-infected tissues. **a** Relative proportions of the Ribo-seq reads aligned to host mRNAs, rRNAs, tRNAs, and 7SL RNAs. **b** The prediction of ribosome heterogeneity in SARS-CoV-2-infected tissues. Each panel corresponds to a dripARF prediction for each indicated time point. All statistical indices were obtained based on dripARF enrichment tests. An enrichment score of 1 represents the normalized enrichment score (NES) of every RP contact point. An enrichment score of 2 corresponds to the deviation of the NES from the background level. An enrichment score 3 indicates whether rRNA positions where rRNA fragment abundance was significantly changed are included in predicted RP contact regions. See also Supplementary Fig. 4a for results obtained 1 dpi and 5 dpi. **c** The crystal structure of the 60 S ribosome large subunit (LSU), wherein 5 S RNP and tRNA are highlighted by different colors. Yellow represents the region in which rRNAs are close to uL5 and uL18 (within 27.4 Å). The coordinates (start and end positions) of the proximal region in rRNA genome sequences (orange: 5 S, cyan: 18 S, and pink: 28 S) are shown in the top square bracket. In heatmaps, white‒black represents distances between RP and rRNA at individual coordinates. The other heatmap with red‒white shows the fold changes in the Ribo-seq reads 7 dpi versus the uninfected controls at the corresponding coordinates. **d** Box plot depicting RPF and RNA level changes in TP53 in lung tissue following SARS-CoV-2 infection.

The elevation in the number of the noncanonical Ribo-seq reads and depletion in the number of RPFs imply that the stoichiometry of conventional ribosomes and other RNP complexes was considerably altered in SARS-CoV-2-infected tissues. In accordance with this finding, the relative proportions of the Ribo-seq reads that were aligned to rRNA, transfer RNA (tRNA), 7SL, and mRNA transcripts exhibited marked changes after viral infection (Fig. 4a). The proportion of 7SL RNA-mapped reads decreased markedly, and the proportions of rRNA and mRNA mapped reads decreased to a lesser extent, suggesting that the number of RNP complexes associating with these RNAs was decreased or that the ability of these RNP complexes to bind partners in the infected tissues was lost.

We found that the expression of ribosomal protein (RP)-coding genes was markedly changed to varying degrees in the infected tissues (Supplementary Fig. 4b and Supplementary Fig. 6d). Different stoichiometries of the constituent proteins may affect the composition of ribosomes. Ribosomes are homogenous complexes; however, it is now known that they form heterogeneous conformations in different biological contexts^34,35^. By analyzing the alignment patterns of Ribo-seq reads across RP contact sites in rRNAs, we investigated whether ribosomes formed distinctive compositions in infected tissues. Intriguingly, the interactions of RP uL5 and uL18 with rRNA were markedly altered in SARS-CoV-2-infected tissues (Fig. 4b and Supplementary Fig. 4a, b). Given that the RNA level of uL5 and uL18 decreased in the late phase, these observations imply that ribosomes in the infected tissues are likely depleted in the RNP complexes comprising the RPs. Notably, uL5 and uL18 are the only RPs that directly interact with 5 S rRNA, constituting 5 S RNP in mature ribosomes.

5 S RNP organizes the functional center of 60 S ribosome subunit, including peptidyl transfer center (PTC) and nascent polypeptide exit tunnel (NPET), and its mutation has been reported to induce flaws in translation elongation, such as compromised fidelity of translational reading frames^36–38^. We identified marked changes in the fragmentation pattern around uL5- and uL8-adjacent regions within rRNAs (Fig. 4c, upper panel). Along with the aforementioned observations, this result indicates at least partially attenuated 5 S RNP associations with the 60 S subunit and 80 S ribosome in SARS-CoV-2-infected tissues. Given that a size reduction and functional center modulation are expected from the deviated 5 S RNP association (Fig. 4c, a lower panel), the ribosome heterogeneity manifesting in SARS-CoV-2 pathogenesis may contribute to the accumulation of the small Ribo-seq reads and affect translation elongation in infected tissues.

The exquisite regulation of 5 S RNP integrity is involved in cell cycle progression and proliferation. It has been reported that the abundant 5 S RNPs activate p53 expression, thereby inducing cell cycle arrest and apoptosis^39,40^. We examined p53 expression in the tissue translatome and transcriptome profiles and found that it was significantly decreased in the late phase (Fig. 4d). The marker genes for cell cycle progression and proliferation (cyclins and histones) were conversely upregulated as the pathogenesis proceeded (Supplementary Fig. 4c, d). These results support the idea that in SARS-CoV-2-infected tissues, ribosome heterogeneity with compromised 5 S rRNP association manifests and even drives the expression of genes related to cell proliferation and survival.

### Ribosomes preferentially stall on transmembrane protein-coding mRNAs in SARS-CoV-2-infected tissues

The potentiated ribosome stalling for the genes with high RAIs and the ribosome heterogeneity with compromised 5 S rRNP association prompted us to look for signs of halted translation elongation in other mRNA transcripts. We identified ribosome stalling sites and assessed their densities based on the A-site information of the canonical Ribo-seq reads (i.e., RPFs) in the tissue Ribo-seq libraries. The ribosome-stalled transcripts exhibited increases in RPF levels to a lesser extent than those in RNA levels, indicating that their translational activities to synthesize proteins were genuinely attenuated (Supplementary Fig. 5a).

**Fig. 5:**
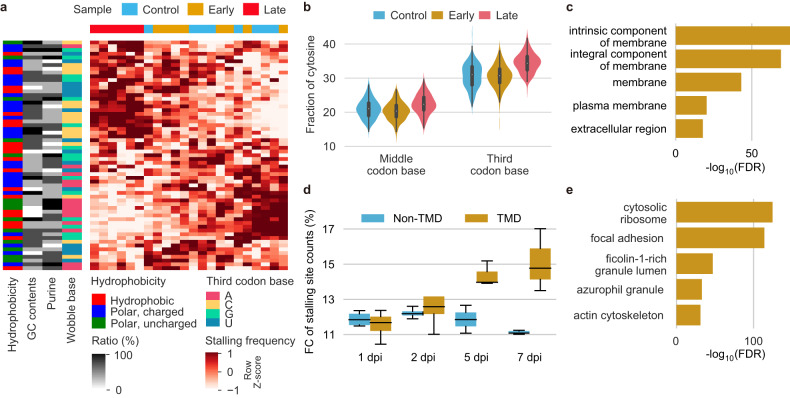
Ribosomes stall preferentially on codons of transmembrane protein-coding mRNAs in the late phase. **a** Heatmap showing the collective intensities of ribosomes stalling on individual amino acid codons. The codon sites with the top 10,000 *Z* scores of RPF A-site enrichment are included in the intensity calculation. The white‒red color key represents a spectrum from the lowest stalling site count (white) to the highest stalling site count (red). Hierarchical clustering based on the Canberra distance between codons (row) or samples (column) was performed (dendrograms not shown). The sidebars show the hydrophobicity group, GC content, purine ratio, and wobble base of each codon. The top color bar indicates the samples. **b** Violin plots representing relative fractions of cytosine in the middle (left) and third base positions (right) of the codons with the top 10,000 Z scores. See also Supplementary Fig. 5b. **c** A bar plot showing GO terms that are overrepresented by genes in which the C frequency in the third base position is high. The bar lengths represent FDR values based on Cohen’s d effect sizes of the associations between the ratios of third base C residues and GO terms. The top five GO terms with the lowest FDRs are shown. **d** Box plot depicting the RPF FC at stalling sites within the transmembrane domain (TMD)- and non-TMD-coding regions throughout the postinfection time course. **e** Gene Ontology (GO) term enrichment analysis using EnrichR revealed the top 1000 genes harboring the strongest intensities of ribosome stalling at 5 and 7 dpi. The top 10 GO terms of cellular components that were significantly enriched (adjusted *P* value < 0.05) are presented.

We examined collective intensities of ribosome stalling per codon at all the time points and found that ribosomes stalled preferentially on a specific subset of codons in the late phase (Fig. 5a). The codons with late phase-specific ribosome stalling showed cytosine enrichment at the third base position (Fig. 5a, b, and Supplementary Fig. 5b). These results suggest that the biased stalling on the “third base C” codons was likely attributable to the preferential pausing of ribosomes on the transcripts that contain these codons at a high frequency. According to GO enrichment analysis, membrane protein-coding genes carry a significantly higher ratio of wobble C codons (Fig. 5c). Despite a similar distribution of codons with the third base C in both transmembrane domain (TMD)- and non-TMD-coding sequences (Supplementary Fig. 5c), the frequency of ribosome stalling was higher in TMD-coding regions than in non-TMD-coding regions (Fig. 5d). These results suggest that the ribosome stalling may be associated with ER (endoplasmic reticulum) membrane-associated complexes, given that translation of TMDs takes place on the membrane of this organelle.

Additionally, GO enrichment analyses of the genes with ribosome stalling demonstrated marked increases in ribosome stalling in the late phase, especially for ribosome- and focal adhesion-associated genes (Fig. 5e and Supplementary Fig. 5d). The profound stalling for ribosome-associated genes likely contributes to the attenuated translation of ribosomal protein-coding genes that are transcriptionally upregulated in the late phase (Supplementary Fig. 6d).

### Attenuated translation of transcriptionally upregulated genes in late SARS-CoV-2 pathogenesis

**Fig. 6:**
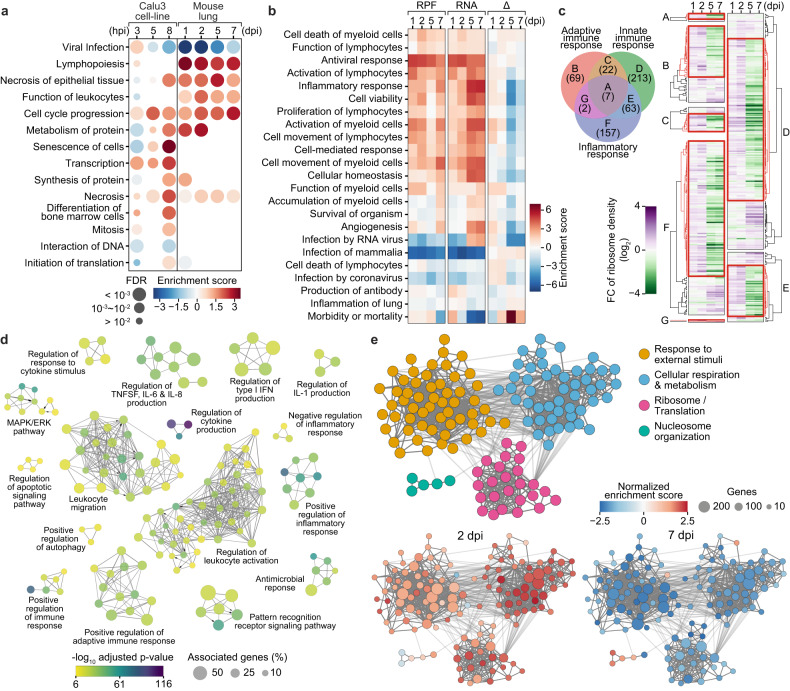
Translation of transcriptionally upregulated genes is compromised in late SARS-CoV-2 pathogenesis. **a** Function and pathway enrichment analysis with RPF level changes of individual genes at each time point post SARS-CoV-2 infection. Comparison between the datasets of mouse lung tissues and human cell lines^26^. The dot sizes and colors indicate the FDRs and enrichment scores, respectively, of individual functions. **b** Heatmaps showing enrichment scores of individual function and pathway terms that were overrepresented in the analyses of RPF and RNA level changes (the left and center panels, respectively) for individual genes in the mouse tissue datasets. The right panels represent the differences between the RPF and RNA enrichment scores of the corresponding terms. **c** Left: a Venn diagram of the genes constituting immune response-related GO terms. Right: Heatmaps showing the temporal changes in ribosome densities (RDs) of the immune response-associated genes in the lung tissues during SARS-CoV-2 pathogenesis. The gene clusters with RD increases 1–2 dpi and decreases 5–7 dpi are indicated by red lines. **d** GO enrichment networks of the immune response-associated genes that exhibited translational downregulation in the late phase and are indicated by red lines in **c**. **e** Networks of the GO terms that were overrepresented in the GSEA of 2 dpi (lower left) and 7 dpi (lower right) groups versus the respective control groups. The dot sizes and colors indicate the number of genes that led to enrichment in individual terms and the normalized enrichment scores, respectively.

The canonical Ribo-seq reads (RPFs) and the RNA-seq reads constituted distinct profiles of temporal gene expression during SARS-CoV-2 pathogenesis in a translation-dependent and translation-independent manner, respectively (Supplementary Fig. 6a). We examined the difference between gene expression changes in the tissues and cell lines after SARS-CoV-2 infection by comparing tissue and cell line translatome profiles. The up- or downregulated genes in the SARS-CoV-2-infected tissues and cell lines negligibly overlapped with each other (Supplementary Fig. 6b), suggesting that the gene regulation or disruption in the tissue during SARS-CoV-2 pathogenesis may be dissimilar with those in the infected cell lines. The predicted phenotypes from function and pathway enrichment analyses corroborated the discrepancy (Fig. 6a). Except for cell cycle progression and necrosis, all the predicted biological functions and pathways in the tissue and cell line translatomes were different.

We performed comparative analyses to anticipate phenotypes from the gene expression changes observed in the tissue Ribo-seq and RNA-seq datasets after SARS-CoV-2 infection (Fig. 6b, Supplementary Table 3 and Supplementary Table 4). The predicted phenotypes and their regulatory directions (positive or negative) were mostly shared between contemporary translatomes and transcriptomes, and immune response-associated terms such as migration and activation of lymphocytes were considerably enriched. However, as for the extent of the predicted phenotype bias (enrichment score), the transcriptomes had more positive values for many terms than the translatomes in the late phase. This observation led us to hypothesize that the increases in the expression of phenotype-associated genes in the translatomes may not catch up with those in the transcriptomes, i.e., translational activities for the transcriptionally upregulated genes may be compromised in the late phase. To examine this hypothesis, we assessed the changes in ribosome densities (RDs) for the annotated genes of immune response GO terms. The majority of the inflammatory, adaptive immune, and innate immune response-associated genes exhibited RD increases and decreases in the early and late phases, respectively (Fig. 6c). The RNA levels of the genes markedly increased in the late phase to a greater extent than their RPF counterparts (Supplementary Fig. 6c). These results imply that compromised translation possibly alleviates the effects of transcriptional upregulation for the immune response genes.

Since not all the annotated immune genes exhibited translationally attenuated gene expression in the late phase, we isolated the subset of genes with the expression pattern (Fig. 6c, demarcated by red lines) and then reconstructed molecular function and pathway networks based on them (Fig. 6d; Supplementary Table 5). Interestingly, multiple pathways, such as Interleukin-1 (IL-1), type 1 interferon, and IL-6 signaling pathways, which are associated with COVID-19 pathology^41,42^, were highlighted. Leukocyte migration-, activation-, and inflammatory response-related terms were underscored as well, suggesting that compromised translation for the immune-related genes may be involved in the immune imbalance in SARS-CoV-2-infected tissues.

To explore other cellular functions and molecular pathways that are modulated by translation regulation or perturbation, we performed GSEA based on the RDs of individual genes. We discovered that a number of genes associated with “response to external stimuli”, “cellular respiration and metabolism”, and “ribosome and translation” terms were translationally upregulated in the early phase and downregulated in the late phase of SARS-CoV-2 pathogenesis (Fig. 6e; Supplementary Table 6), suggesting that regulation in each direction may play dominant roles in early and late SARS-CoV-2 pathology. The RP-coding genes that exhibited clear ribosome stalling in the late phase were highlighted here as well. We examined the changes in RPF and RNA levels of canonical RP-coding genes (Supplementary Fig. 6d), and the results revealed bipartite regulatory pathways of basic translation machinery constituents during pathogenesis. In the early phase, the RNA levels of the RP-coding genes markedly decreased despite their enhanced translation, while in the late phase, the genes were transcriptionally activated, but their translation was strongly compromised. Intriguingly, mitochondrial ribosome protein-coding genes were maintained at relatively constant levels of expression both in the translatomes and transcriptomes. These observations indicate that disrupted production of canonical ribosomes in respiratory tissues inevitably ensues from SARS-CoV-2 infection.

### Mouse homologs of human COVID-19 signatures exhibit attenuated translation activities

**Fig. 7:**
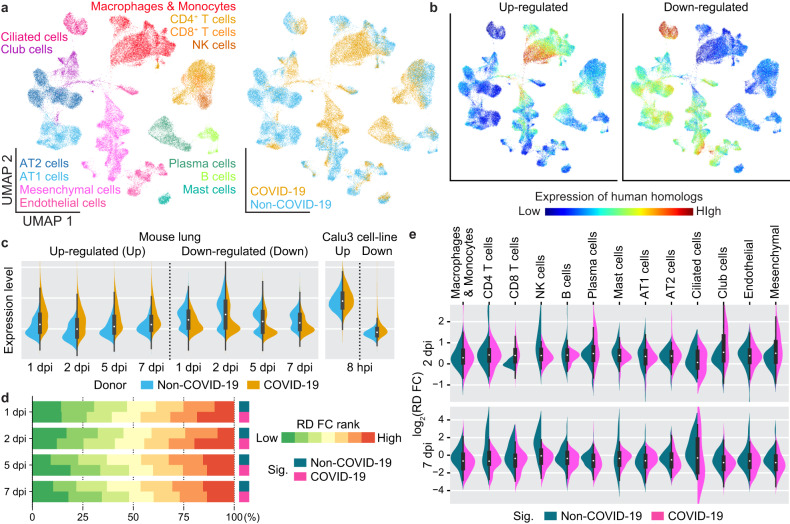
Compromised translation of COVID-19 signature genes in mouse tissues. **a** UMAP visualization of the single-cell transcriptome from lung samples taken during the autopsies of COVID-19 and non-COVID-19 patients^4^. The datasets from the patients with acute symptoms and early deaths (5–16 days from symptom appearance to death) were selected and re-analyzed. Cell types (left) and origins (right) are indicated by different colors. **b** Single-cell expression of human homologs of the up- (left) and downregulated (right) genes of the mouse lung translatome 7 dpi. See also Supplementary Fig. 7a for results obtained 1, 2, and 5 dpi. **c** Collective expression of human homologs of the up- or downregulated genes of the mouse tissue translatomes 1, 2, 5, and 7 dpi (left) and of the human cell line translatome (right) in non-COVID-19 (blue) and COVID-19 (orange) donor group 8 dpi. **d** Discretized bar plots showing comparisons between RD FCs of mouse homologs of collective non-COVID-19 and COVID-19 signatures at individual time points postinfection. **e** Violin plots showing the RD FCs of mouse homologs of cell type-specific non-COVID-19 and COVID-19 signatures. RD FC obtained 2 dpi and 7 dpi are shown.

To examine the homology between gene regulation in our mouse model of SARS-CoV-2 pathology and human COVID-19 patients, we exploited single-cell transcriptomes of lung autopsy tissues from COVID-19 and non-COVID-19 donors (Fig. 7a)^4^. We assessed single-cell expressions for human homologs of the up- and downregulated genes in the mouse translatomes after SARS-CoV-2 infection. Uniform manifold approximation and projection (UMAP) plots indicated that the up- and downregulated genes were predominantly expressed in COVID-19 and non-COVID-19 donor cells, respectively (Fig. 7b and Supplementary Fig. 7a). The homologs of the upregulated genes were most prominently expressed in macrophages and monocytes, which is a reasonable outcome given that SARS-CoV-2 infection induces massive infiltration of innate immune cells into mouse and human lungs. In addition to these results, the collective expressions for the human homologs of the up- and downregulated genes in SARS-CoV-2-infected mouse tissues exhibited higher expression in COVID-19 and non-COVID-19 populations, respectively (Fig. 7c, the left panel), indicating that gene expression changes in human and mouse lungs with SARS-CoV-2 pathology are at least partially similar. The up- and downregulated gene sets in the human cell line translatomes showed less similar and, in some cases, opposite patterns (Fig. 7c, a right panel), which, paradoxically, supports the relevance of the mouse tissue translatome in recapitulating gene regulation in human COVID patients.

We identified marker genes for the cell type clusters based on the human single-cell transcriptomes and examined RDs of their respective mouse homologs (Supplementary Fig. 7c). None of the marker gene sets were predominant among those with RD decreases in the late phase, indicating that the compromised translation in the infected tissues was unlikely to be exclusive to certain cell types. We then isolated COVID-19 and non-COVID-19 signature genes that had been exclusively expressed by each donor in every cell type cluster (Supplementary Fig. 7b; Supplementary Table 7). Intriguingly, the RDs of the COVID-19 signatures decreased in the late phase to a greater extent than those of their non-COVID-19 counterparts (Fig. 7d). This result implies that attenuated translation may preferentially occur for a common set of genes that are transcriptionally upregulated after SARS-CoV-2 infection in both species. We then examined RD changes in the signatures of individual cell types (Fig. 7e). RD decreases of COVID-19 signatures in the late phase showed preponderances for most immune cells, except B cells and mast cells, suggesting that translation might be preferentially compromised in these cells. In the early datasets, the COVID-19 signatures of CD8 + T cells, plasma cells, club cells, and mesenchymal cells exhibited larger RD increases, alluding to synergy between translational and transcriptional upregulation in these cells during the early pathogenesis.

## Discussion

What and how viral and host genes are regulated in virus-infected tissues are important aspects of the pathophysiology of all viral diseases, including COVID-19. Given that there is currently no translatome constructed from any virus-infected tissue, our study offers a unique resource to directly study gene translation in vivo within tissues that undergo viral pathogenesis as well as SARS-CoV-2 pathogenesis.

The translation and transcription of SARS-CoV-2 virus in tissues were very different from those in the cell lines, highlighting unseen actions of the virus at the molecular level in physiological contexts. In particular, the relatively low levels of sgRNAs and subdued expansion of SARS-CoV-2 imply that the host defense system may directly impede sgRNA synthesis of RNA-dependent RNA polymerase (RDRP) or eliminate infected cells where sgRNAs are actively produced. Given that the proportions of M and N protein-coding sgRNAs were markedly lower in the tissues versus the cell lines, either scenario may probably work when the expression of these viral genes is high. It will be intriguing to isolate the temporal RDRP complexes from infected tissues and identify the associated proteins, which may reveal the hidden defense mechanisms that disrupt viral expansion.

Unexpectedly, our analysis of the tissue Ribo-seq libraries uncovered hitherto-unidentified RNP interactions in SARS-CoV-2-infected tissues. No previous translatome studies on virus-infected cell lines had reported this phenomenon, implying that exclusive or specific factors in the tissue microenvironment are likely to be key triggers. Given that RPF abnormalities continuously accrued even after SARS-CoV-2 infection is attenuated in tissue (5 and 7 dpi), their emergence is unlikely to be dependent on viral activity. The pseudoribosomal RNP interactions were coincidentally identified in conjunction with specific pathological features, such as extensive immune cell infiltration and pulmonary structure collapse (Supplementary Fig. 1c, d), which may allude to causal relationships between RBP accumulation and pathological events. It will certainly be interesting to investigate whether excessive immune activation or tissue damage leads to the production of noncanonical Ribo-seq reads. If it does, then RNP interactions may be critical signatures in diverse pathological contexts, including other acute and severe respiratory disease contexts. Similarly, RPF abnormalities more likely originate from endogenous RNP complexes that form during pathogenesis rather than exotic viral complexes. To clarify the molecular origin of the pseudoribosomal RNP interactions, isolating and identifying the RNP complexes in SARS-CoV-2-infected tissue is essential and may elucidate a novel molecular pathway that explains the gene regulation or perturbation underlying SARS-CoV-2 pathology.

The positive correlation between RPF abnormality and RNA level increases of individual genes suggests that the pseudoribosomal RNPs may preferentially target newly and actively synthesized RNAs. In addition, the decreased densities and the elevated pausing rates of ribosomes for the genes with the RPF abnormality increases suggest that the RNPs and canonical ribosomes may conceivably compete for access to newly transcribed transcripts. Given that translation of the transcriptionally upregulated genes is compromised during late pathogenesis, SARS-CoV-2-infected tissues may hardly attain the required amounts of certain gene products (i.e., proteins) to recover from damage caused by the viral invasion, regardless of the transcriptional reprogramming that induces the expression of these genes. If this is the case, then resolving the RBP complexes may be a key strategy for mitigating SARS-CoV-2 pathogenesis.

Our study proposes that disruption of RNP complexes that are associated with translation is one of the most prominent molecular events underlying SARS-CoV-2 pathology. The dichotomous suppression of RP-coding genes is thought to reduce ribosome steady-state levels and global translational capacities in infected tissues. Moreover, the differential magnitudes of RP gene downregulation are linked to ribosome heterogeneity. The relatively low expression of RP uL5 and uL18 may result in attenuated 5 S RNP associations with large ribosomal subunits, which may induce defects in translation elongation. The RPF abnormalities may originate from heterogeneous ribosome–RNA associations, although further study is required to verify this scenario. In addition, the potentiated ribosome stalling in TMD-coding regions alludes to disturbed elongating ribosome complexes that are associated with ER. Decreases in 7SL RNA-mapped Ribo-seq reads may be linked to unstable ER-associated mRNA translation. The molecular mechanisms underlying both the hindrances to translation elongation are currently unclear, but they will certainly be intriguing areas to investigate.

### Supplementary information

Supplementary Information

Supplementary Table 1

Supplementary Table 2

Supplementary Table 3

Supplementary Table 4

Supplementary Table 5

Supplementary Table 6

Supplementary Table 7

## Data Availability

All raw sequencing data files were deposited in the Gene Expression Omnibus under the accession number GSE222252.
