# Author Correction: SARS-CoV-2 infection engenders heterogeneous ribonucleoprotein interactions to impede translation elongation in the lungs

**DOI:** 10.1038/s12276-023-01134-6

**Published:** 2023-12-25

**Authors:** Junsoo Kim, Daehwa Youn, Seunghoon Choi, Youn Woo Lee, Dulguun Sumberzul, Jeongeun Yoon, Hanju Lee, Jong Woo Bae, Hyuna Noh, Dain On, Seung-Min Hong, Se-Hee An, Hui Jeong Jang, Seo Yeon Kim, Young Been Kim, Ji-Yeon Hwang, Hyo-Jung Lee, Hong Bin Kim, Jun Won Park, Jun-Won Yun, Jeon-Soo Shin, Jun-Young Seo, Ki Taek Nam, Kang-Seuk Choi, Ho-Young Lee, Hyeshik Chang, Je Kyung Seong, Jun Cho

**Affiliations:** 1 Center for RNA Research, Institute for Basic Science (IBS), Seoul National University, Seoul, Republic of Korea; 2 Interdisciplinary Program in Bioinformatics, Seoul National University, Seoul, Republic of Korea; 3 Department of Biomedical Science and Engineering, Gwangju Institute of Science and Technology (GIST), Gwangju, Republic of Korea; 4 Korea Mouse Phenotyping Center (KMPC), Seoul National University, Seoul, Republic of Korea; 5 Laboratory of Developmental Biology and Genomics, Research Institute for Veterinary Science, and BK 21 PLUS Program for Creative Veterinary Science Research, College of Veterinary Medicine, Seoul National University, Seoul, Republic of Korea; 6 Department of Nuclear Medicine, Seoul National University Bundang Hospital, Seongnam, Republic of Korea; 7 School of Biological Sciences, Seoul National University, Seoul, Republic of Korea; 8 Laboratory of Avian Diseases, Research Institute for Veterinary Science, and BK 21 PLUS Program for Creative Veterinary Science Research, College of Veterinary Medicine, Seoul National University, Seoul, Republic of Korea; 9 Preclinical Research Center, Seoul National University Bundang Hospital, Seongnam, Republic of Korea; 10 Department of Periodontology, Section of Dentistry, Seoul National University Bundang Hospital, Seongnam, Republic of Korea; 11 Department of Internal Medicine, Seoul National University Bundang Hospital, Seoul National University College of Medicine, Seongnam, Republic of Korea; 12 Division of Biomedical Convergence, College of Biomedical Science, Kangwon National University, ChunCheon, Republic of Korea; 13 Laboratory of Veterinary Toxicology, College of Veterinary Medicine, Seoul National University, Seoul, Republic of Korea; 14 Severance Biomedical Science Institute, Graduate School of Medical Science, Brain Korea 21 Project, Yonsei University College of Medicine, Seoul, Republic of Korea; 15 Department of Nuclear Medicine, Seoul National University, College of Medicine, Seoul, Republic of Korea; 16 Interdisciplinary Program and BIO MAX Institute, Seoul National University, Seoul, Republic of Korea

**Keywords:** Ribosome, Viral infection

Correction to: *Experimental & Molecular Medicine* 10.1038/s12276-023-01110-0, published online 01 November 2023

After online publication of this article, the authors noticed an error in the description of the authorship, which could mislead readers to think all the co-first and co-corresponding authors contributed equally to the study regardless of their respective positions.

This corrigendum is to note that Junsoo Kim, Daehwa Youn, and Seunghoon Choi should have been acknowledged as co-first authors, and Ho-Young Lee, Hyeshik Chang, Je Kyung Seong, and Jun Cho should have also been listed as co-corresponding authors on the paper.

The affiliation details for Jong Woo Bae were incorrectly given as ‘2 Interdisciplinary Program in Bioinformatics, Seoul National University, Seoul, Republic of Korea’ but should have been ‘7 School of Biological Sciences, Seoul National University, Seoul, Republic of Korea’.

The authors apologize for any inconvenience caused.

The original article has been corrected.

