# Supplementary material for: SARS-CoV-2 infection engenders heterogeneous ribonucleoprotein interactions to impede translation elongation in the lungs
Source: Exp Mol Med. 2023 Nov 1;55(12):2541–52. doi: 10.1038/s12276-023-01110-0 (PMC10767024; doi:10.1038/s12276-023-01110-0)
Supplement: Supplementary file 1 — Supplementary Information [file 12276_2023_1110_MOESM1_ESM.pdf]

Supplementary Figures

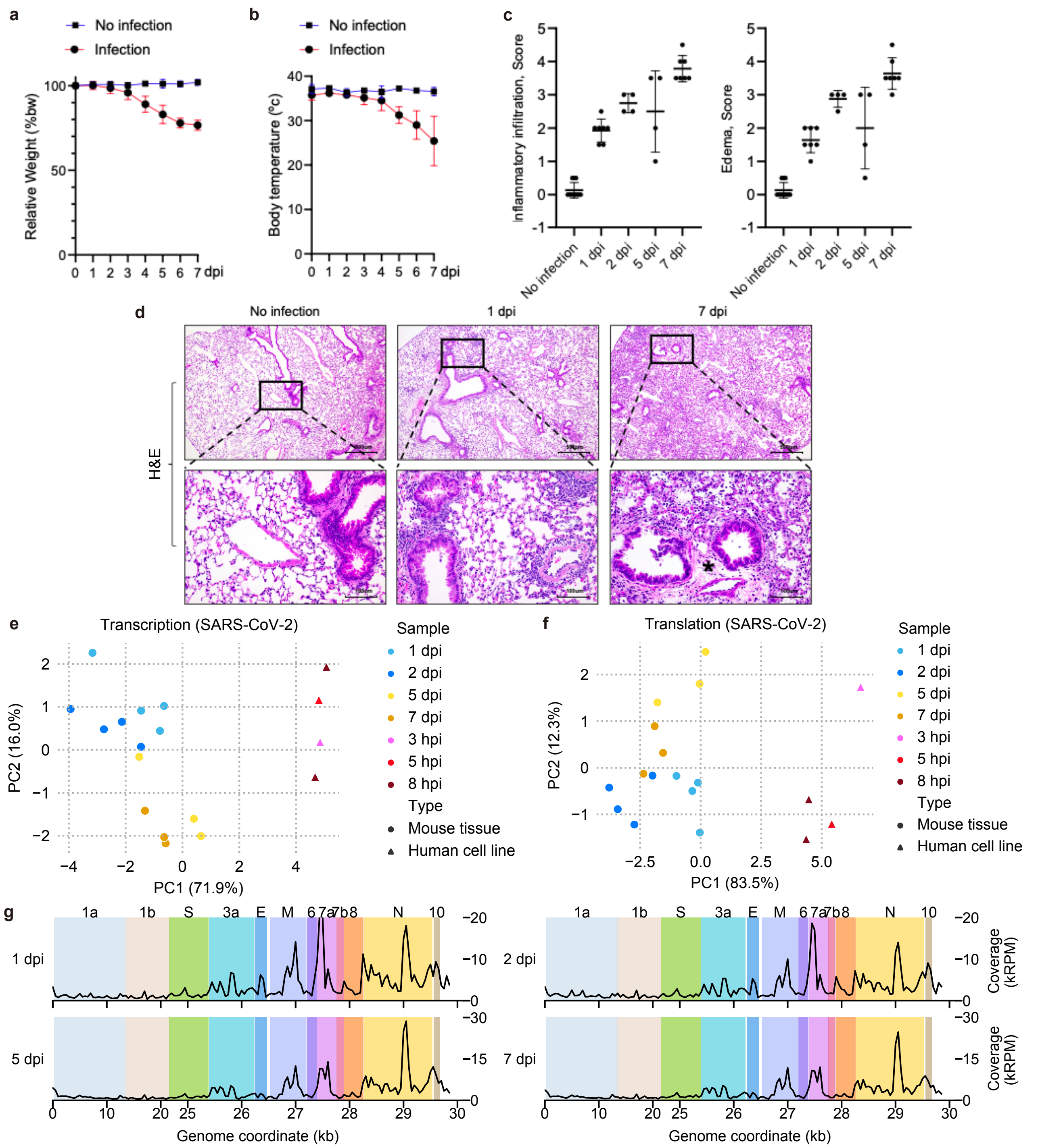

Supplementary Fig. 1 Viral gene expressions depicted in the tissue translatomes and transcriptomes. a Weight changes in the mice. Body weights (BW) at each dpi were normalized by the BWs of uninfected mice at 0 dpi (%). **b** Body temperature changes in the mice. **c** Histopathological scores of lung inflammatory infiltration and edema were assessed by H&E staining. **d** Illustrations of H&E staining of lung tissues. Asterisks indicate perivascular edema. **e, f** PCA of the viral transcriptomes (**e**) and transcriptomes (**f**). The color specifies the sample group, and the marker type represents the source of the dataset. Human cell line data was obtained from Finkel, et al. **g** Read densities of the Ribo-seq libraries across the SARS-CoV-2 genome.

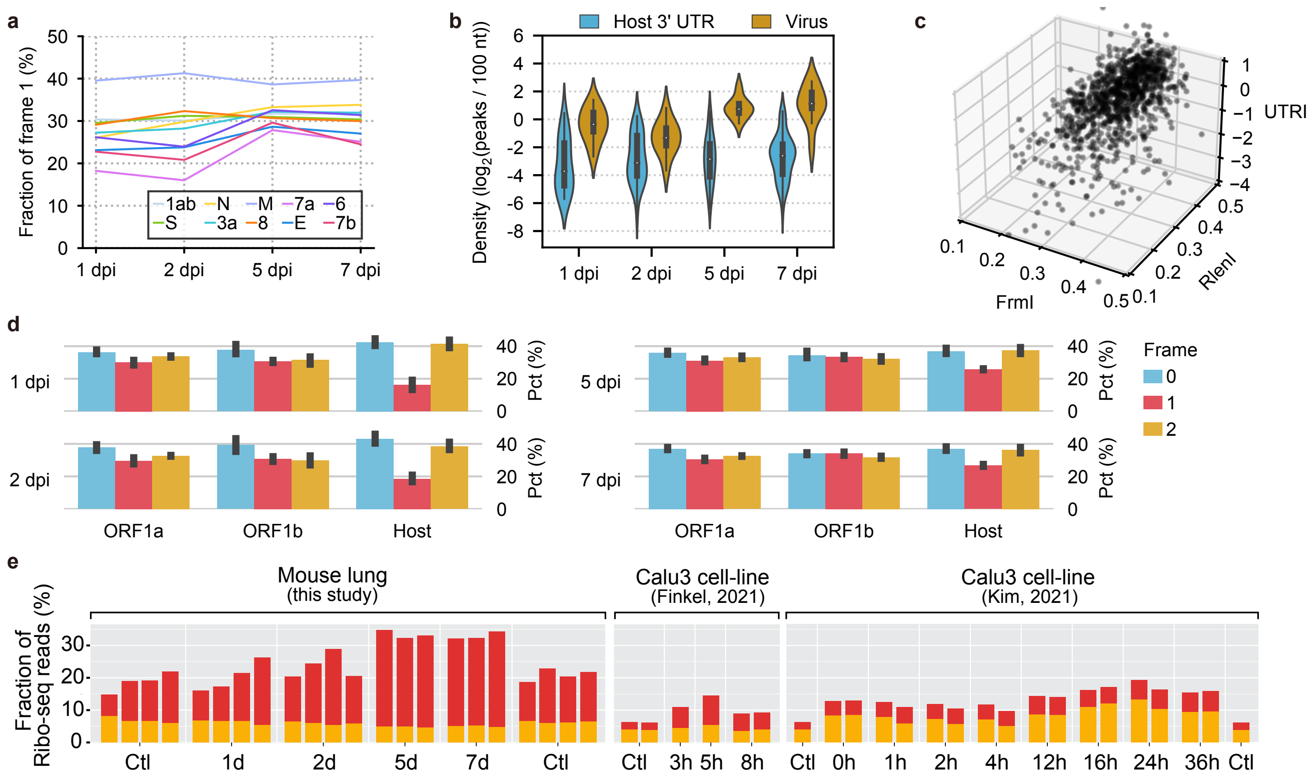

Supplementary Fig. 2 Non-canonical Ribo-seq reads emerge in the tissue Ribo-seq upon SARS-CoV-2 infection. a A relative fraction of the Ribo-seq reads mapped to frame 1 of all codons in viral transcripts. **b** Densities of the Ribo-seq read peaks in 3′ UTR of host mRNA and CDS of viral transcript. Piranha was used to identify genomic regions of statistically significant read enrichment (Ribo-seq read peaks). The top 1,000 transcripts with the highest UTRI were chosen for the host. **c** Comparison of three RAIs. A 3D scatter plot of randomly selected 1,000 genes using FrmI (the relative ratio of frame 1) and RlenI (the relative ratio of short RPF) as the X and Y axes, respectively. The Z axis represents the UTRI (the relative ratio of RPF mapped to the 3′ UTR compared to the CDS). **d** Ratio of the Ribo-seq reads mapped to each frame in ORF1a, ORF1b, and host transcripts. Error bars reflect the standard deviation of biological replicates. **e** Ratios of 3′ UTR and 5′ UTR aligned reads in the Ribo-seq libraries of mouse lung tissues and human cell lines, an extended presentation of Fig. 2b.

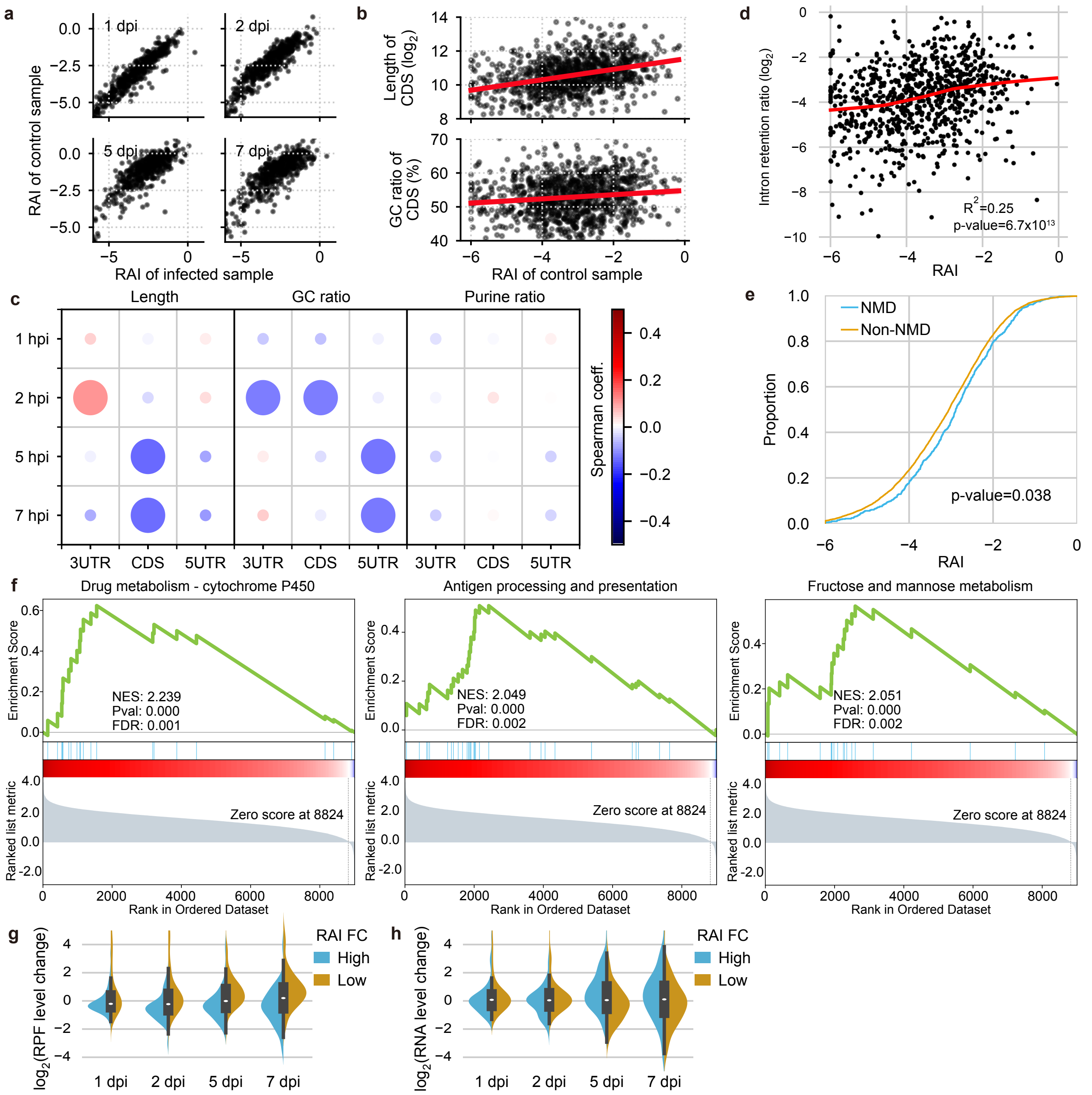

Supplementary Fig. 3 The multiple origins for the non-canonical Ribo-seq reads. a Scatter plots of the RAI levels in the infected and control samples. 500 randomly chosen transcripts are shown for better visibility. **b** A scatter plot of RAI level in the control sample against the length of CDS (top) and the GC ratio of CDS (bottom). The red lines represent trend lines constructed by the point distributions. For a better visibility, 1000 randomly chosen transcripts are shown. **c** A heat map showing the Spearman correlation coefficients of pairwise comparisons between RAI index FC and sequence properties. The color gradient and size represent the coefficient and p-value, respectively. **d** Correlation analysis of RAI and intron retention ratios. The red line represents a trend line constructed by the point distributions. The intron retention values are the median depth of the relevant intronic region as measured by IRFinder. **e** Cumulative distribution of RAIs of nonsense-mediated mRNA decay (NMD) and non-NMD genes. The list of NMD genes is obtained from Ensembl 104. p-values, one-sided Mann-Whitney U tests. **f** GSEA enrichment plot (KEGG pathway) of the top three significantly enriched terms derived from comparisons of RAIs at 5 or 7 dpi and their respective controls. **g, h** Violin plots indicating the changes in RPF (**g**) or RNA (**h**) levels of the genes with high and low RAI FCs.

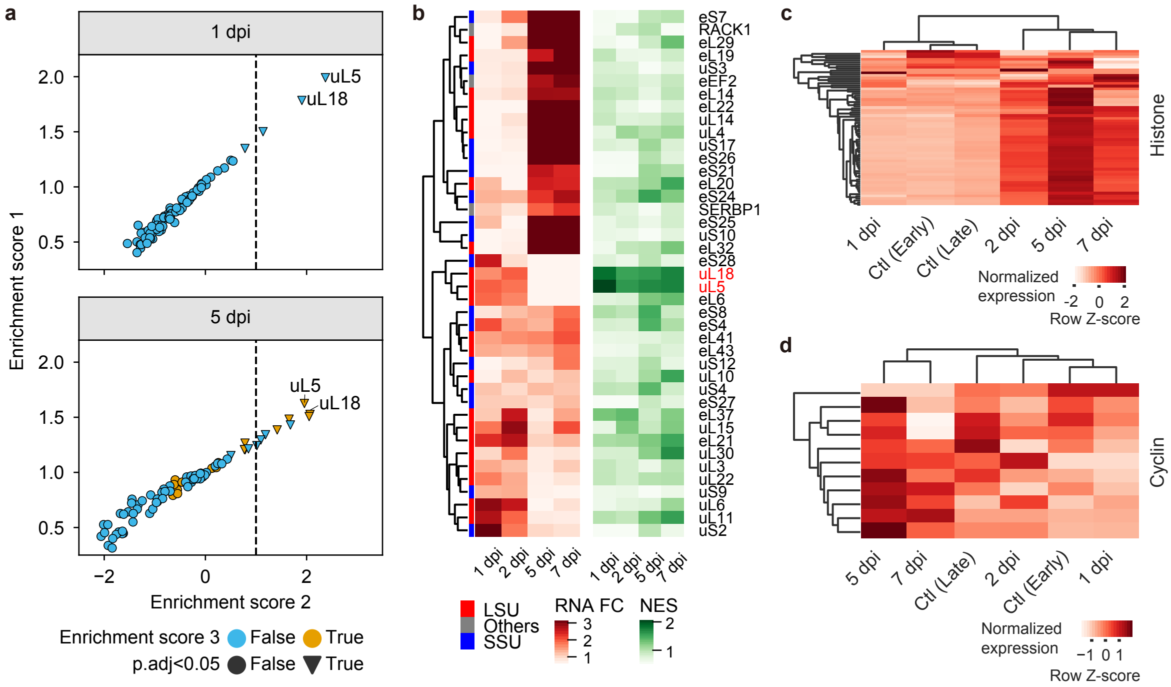

Supplementary Fig. 4 Altered RP-rRNA interactions within ribosomes during SARS-CoV-2 pathogenesis. a The prediction of ribosome heterogeneity in the SARS-CoV-2-infected tissues. Each panel corresponds to a dripARF prediction at each indicated time point. All statistical indices are obtained from the enrichment tests of dripARF. Enrichment score 1 represents the normalized enrichment score (NES) of every RP contact point. Enrichment score 2 corresponds to its deviation from background levels. Enrichment score 3 indicates whether rRNA positions where rRNA fragment abundance was significantly changed are included in predicted RP contact regions. See also Fig. 4b for results at 2 dpi and 7 dpi. **b** Heat maps showing RNA expression changes (left) and predictive contribution to ribosome heterogeneity (right) of individual RPs at all the time points of the post-SARS-CoV-2 infection. **c, d** Heat maps showing the changes in RNA expression levels for histone (**c**) and cyclin (**d**) genes. Expression values for each gene (row) are normalized across all samples (columns) by z-score calculation.

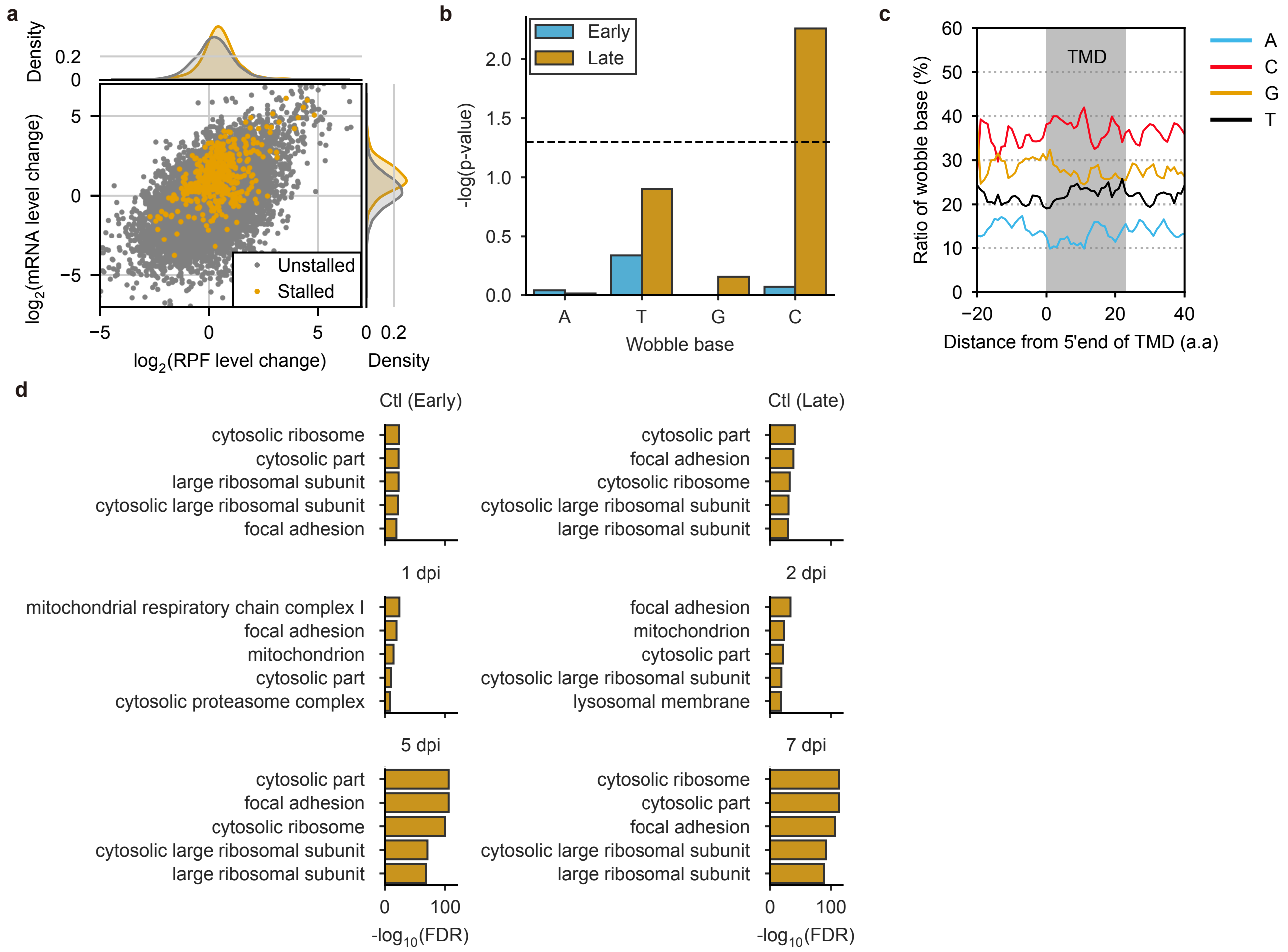

Supplementary Fig. 5 Ribosome stalling predominantly occurs on the codons with cytosine at the third base position. a A scatter plot of RPF level changes versus mRNA expression changes of individual genes. The marginal density plots show the distribution of scatters for each axis. Genes harboring more than one single stalling site (yellow) are classified as a "stalled" group. **b** A bar plot of the Wilcoxon rank-sum test for the changes in stalled RPF levels with "A", "T", "G", or "C" at the third base of codons. **c** A line plot displaying the mean ratios of the third base of codons around TMD-coding regions. The representative TMD-coding region (highlighted with a grey background) has the mean size of all the TMD-coding regions. **d** GO term enrichment analysis for the top 1000 genes with the strongest ribosome stalling. The top five cellular components GO terms that are most significantly enriched (adjusted p-value < 0.05) are displayed.

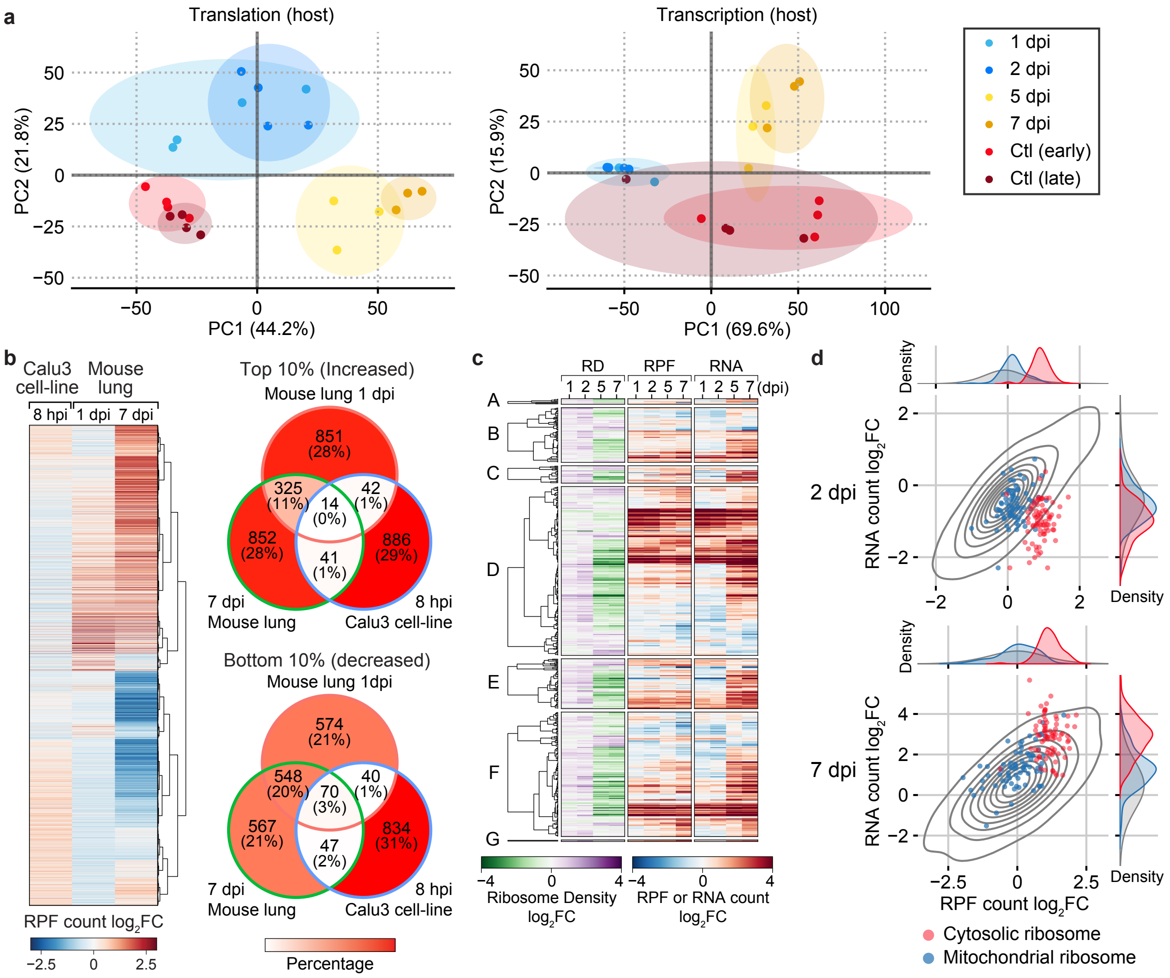

Supplementary Fig. 6 Function and pathway enrichment analyses of transcriptomes and translatomes of SARS-CoV-2-infected tissues. a PCA of the host translatomes (left) and transcriptomes (right). PCA coordinates were determined by the RPF and RNA levels of individual genes in each library, respectively. The colors indicate the sample groups. **b** Comparisons between the up- or down-regulated genes identified from the translatomes of mouse tissue and human cell line. The heatmap (left) represents the changes in RPF levels at 8 hpi (human) and at 1 and 7 dpi (mouse). The Venn diagrams (right) indicate how many genes are overlapped. **c** Heatmaps showing the changes in ribosome density, RPF, and RNA level of immune response-associated genes, related to Fig. 6c. **d** Scatter plots of the RPF and RNA level changes at 2 and 7 dpi. Cytosolic ribosome-related and mitochondrial ribosome-related genes are highlighted with red and blue dots, respectively.

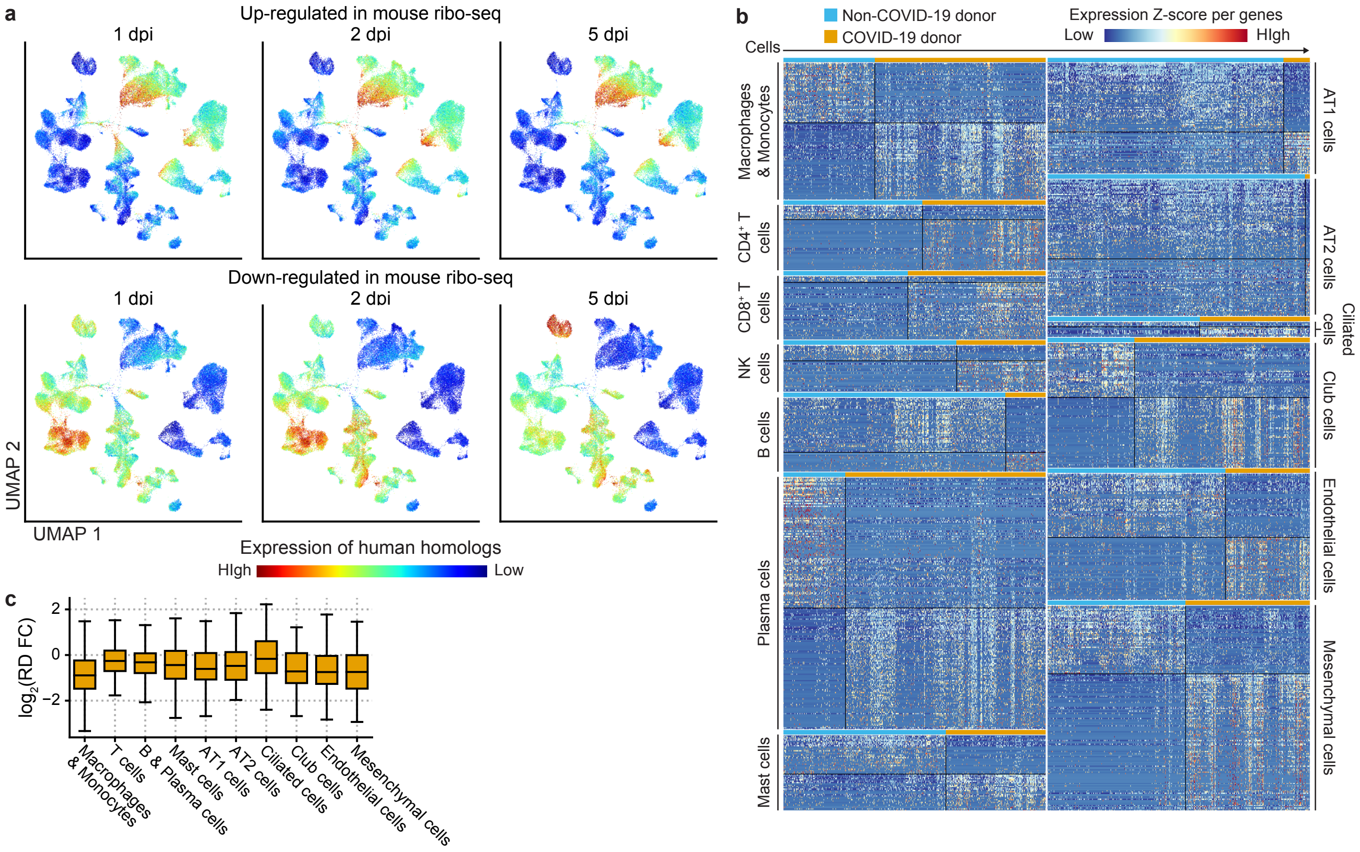

Supplementary Fig. 7 Comparative analyses of mouse translatome with human COVID-19 single-cell transcriptome. a Single-cell expressions of human homologues of the up- (top) and down-regulated (bottom) genes at 1, 2, and 5 dpi of the mouse tissue translatomes. **b** Heat maps showing single-cell expression of the COVID-19 and non-COVID-19 signatures. Each heat map represents the result of each cell type determined in the analysis of Fig. 7a. Rows are grouped into non-COVID-19 signatures (upper) and COVID-19 signatures (lower) that exhibit exclusive expressions to non-COVID-19 donors (left, blue) and COVID-19 donors (right, orange), respectively. **c** Boxplots indicating the changes in RDs for the marker genes of the cell types that were determined in the analysis of Fig. 7a.

Supplementary Methods

Histopathology

The lung tissues of mice were fixed in neutral buffered 10% formalin for 1 day and processed using a standard method. Hematoxylin and eosin (H&E) were used to stain the 3-μm paraffin-embedded sections. The lesions were graded using a semi-quantitative scale based on the percentage of tissue affected by pulmonary inflammation as follows: 0, absent; 1, minimal, < 10% of tissue affected; 2, mild, ≧ 10% and < 25% of tissue affected; 3, moderate, ≧ 25% and < 50% of tissue affected; 4, moderately severe, ≧ 50% and < 75% of tissue affected; or 5, severe, ≧ 75% of tissue affected. The presence and abundance of lesions such as inflammatory cell infiltration and edema were used to determine the severity of pulmonary inflammation. The presence of inflammatory cell infiltration was defined as lesions involving the perivascular and peribronchial spaces with a moderate number of inflammatory cells and interstitial spaces with more than five inflammatory cells in each alveolar space. The presence of pulmonary edema was defined as lesions involving the perivascular spaces exhibiting edematous cuffs and alveolar edematous spaces. Histopathological scores were determined by more than two veterinary pathologists.

Pre-processing and alignment for Ribo-Seq and RNA-Seq libraries

For sequenced reads from the Ribo-Seq library, adaptor sequences (Illumina TruSeq RA3) at the 3′ ends were removed, and reads between 20 nt and 40 nt were selected using cutadapt version 3.4 with the following options: "-u 1 --overlap 6 --minimum-length 20 --maximum-length 40"[1]. For RNA-Seq reads, only the forward strand was used for the downstream analysis. Both Ribo-Seq and RNA-Seq reads were each mapped to common noncoding RNAs (ncRNAs), including ribosomal DNA complete repeating units (Rfam accession RF00001, RF00002, RF01960, and RF02543), signal recognition particle DNA (Rfam accession RF00017), and genomic tRNA (GtRNAdb release 19) using HISAT2 version 2.2.1 with the --very-sensitive option. Unmapped reads were aligned to both the mouse genome (GENCODE GRCm39 Release M27) and the SARS-CoV-2 genome (NC_045512.2) by HISAT2 version 2.2.1[2,3]. For the alignment, the following options were used: "--pen-noncansplice 0 --very-sensitive --no-softclip --rna-strandness F -k 32 --secondary". Two samples, one from the 5-day post-infection (dpi) group and another from the 7 dpi group, were excluded from the RPF analyses adopted in Figures 2, 3, 4, and 5. The sample from the 5 dpi group exhibited both physiological and molecular indications of an abortive infection, demonstrated by a negligible remaining viral population in contrast to other replicates. Similarly, a sample from the 7 dpi group displayed quality issues potentially arising from suboptimal sample preparation or recovery during the monosome fractionation process. The diversity in read sequence composition was significantly less for the RPF

reads in this sample, likely due to over-amplification, with its RPF sequence composition fundamentally differing from the rest of the samples.

Filtering and annotation

Using intersectBed of BEDTools and FilterSamReads of Picard, reads that were mapped into the mouse repeat region (according to the UCSC genome annotation database) are eliminated[4]. The following priorities of the GENCODE annotation (version M27) column: “basic”, “CCDS”, and “appris principal” determined a single representative isoform from several mRNA isoforms for each gene. For downstream mRNA analysis, only the reads that were mapped to nuclear-encoded, protein-coding transcripts were included. The 5′ end of reads was used to determine the mapped position on the genome using bedtools genomecov version 2.30.0. According to read length, RPF reads were divided into two groups: 20–29 (short) and 30–34 nt (long). For analyzing the human RNA-Seq and RPF-Seq data from previous studies, the reads mapped to the human repeat region were filtered, and a different version of the GENCODE annotation (version 38) was used[5].

Processing the human datasets from previous studies

The same software pipeline was used to process the RNA-Seq and Ribo-Seq datasets from two earlier studies[5,6] which infected human cell lines with SARS-CoV-2. However, different adaptor sequences, reference genomes (GRCh38), noncoding RNAs, and GENCODE annotation (version 38) that correspond to human were used.

Quantification of SARS-CoV-2 ORF expression level

The quantity of RNA expression for each viral sgRNA was calculated using the RNA-Seq reads that covered the TRS-L and TRS-B junctions. The location of junctions was established using earlier studies[7]. The reads mapped to ORF7a and ORF7b overlapping regions were classified as ORF7a. For the coverage plot on the viral genome, the read depths were binned into 50-nt bins and plotted by using averages to smooth the lines. To calculate the sgRNA to gRNA ratio, the forward stranded RNA-Seq reads (>99 nt) that span the viral genomic region between 50 and 60 nt were divided into the sgRNA and gRNA groups depending on the presence of large deletions (>20,000 nt).

Calculation of RPF abnormality indices

To explain the non-canonical Ribo-Seq read pattern in the late infection phase group, the ratio of reads mapped to the reading frame 1 in the CDS (FrmI), the proportion of reads aligned to the 3′ UTR against the CDS (UTRI), and the percentage of short reads within the CDS (RlenI) were calculated for each transcript. In order to normalize the Ribo-Seq read counts for each transcript region, the lengths of the CDS and 3′ UTR

from the Gencode annotation were employed. UTRI was used as a representative RAI for downstream analysis.

Transcript-level quantification and normalization

In order to compute the distance of each Ribo-Seq read from start or stop codons, the CDS region in the GENCODE annotation was used. Transcripts that have identical start and end codon positions in the annotation file are discarded. Ribo-Seq read counts were normalized in RPM and averaged across multiple replicates. For the genomic region distribution plot, a modified annotation file with the positions of start and stop codons shifted by 25 nt towards 5′ ends was used, and the frequencies of Ribo-Seq mapped in the 5′ UTR, CDS, and 3′ UTR regions of protein-coding genes were determined. For host gene metagene analysis, the top 1,000 highly translated genes were chosen, and the proportions of Ribo-Seq reads mapped to each genomic position were calculated.

Determining the wobble base at the ribosomal A-site

Using integer programming, the A-site offset from the 5′ end for each read length was calculated[8]. For reads that are 30-33 bp, the offsets are +15. Each RPF's reading frame was identified using the GENCODE annotation's canonical start codon location. Filtered out were RPFs that corresponded to either non-coding transcripts or UTR regions. A wobble base at the ribosomal A-site was determined from the nucleotide at the third position of the corresponding codon.

Measuring the distance between RPF peaks

To identify the regions with substantial enrichment for RPF reads in the host 3′ UTR and viral transcripts, peak-calling software, Piranha version 1.2.1, was used[9]. For the purpose of choosing the significant peaks, the FDR cutoff of 0.05 was used. For each sample, the distance between the peaks in the same transcript was measured and then averaged.

PCA analysis of RAI across host genes

StandardScaler and T-SNE (perplexity = 50) from scikit-learn version 0.24.2 were used to scale and analyze the RAI of host genes for principal component analysis[10]. Each gene's subcellular localization was obtained from the UniProtKB database (retrieved from uniprot.org in December 2021)[11]. The UniProt ID mapping table was used to map UniProtKB accession numbers to the Ensembl mRNA accession ID.

Enrichment analysis

To identify enriched GO terms associated with high RAI genes in the control group, the genes with a mean RAI of above 0.5 in the control sample were analyzed for

EnrichR using GSEApy version 0.10.8. Cellular component terms with a P-adjusted value below 0.05 were deemed significant. For identifying the pathways enriched in high RAI change, GSEA was performed using the pre-ranked module (permutation_num = 100,000). KEGG (version 2016) terms with a P-adjusted value below 0.05 were chosen. To avoid redundancy among the top ten terms, those with fewer than 15 gene sets and more than 30% overlapping genes are excluded. To examine the GO terms that are enriched with genes having a high cytosine ratio at the wobble position, the proportion of wobble C among specific GO terms compared to the background genes was examined by using Cohen's d test. Each FDR was calculated by the Benjamini-Hochberg method. To overcome the gene-set redundancy problem, the gene set in each parent node was changed to include all the gene sets in the child nodes.

To identify GO terms associated with genes with high ribosome density changes, GO enrichment analysis is performed using GSEApy, comparing the ribosome density values of each time point with their corresponding controls. For the analyses, information on GO terms with the level between 4 to 6 is selected from the gene set database "m5.go.v2022.1.Mm.symbols.gmt" provided by MSigDB[12,13]. The GSEA module was applied with the following options; minimum gene set size is 15, maximum gene set size is 500, permutation type is 'phenotype', the number of permutations is 3000, and the used method is 'signal_to_noise'. To generate the enrichment networks, GO terms that have the top 50 or bottom 50 normalized enrichment score and statistical significance (p-value < 0.05) in at least one condition are selected, and then 1000 connections between selected GO terms that have the highest Sørensen–Dice coefficient are shown as network with Cytoscape v3.9.1[14].

To identify what detailed function the immune response-associated genes that exhibited translational down-regulation in the late phase are associated with, ClueGO v2.5.9[15] and Cytoscape are used to generate the GO enrichment network with the following options; GO terms with level 5 are selected, terms that include at least 15 and 8% genes are enriched and connections between terms with Cohen's kappa coefficient > 0.4 are presented as the network.

Calculation of intron retention ratio

Using IRFinder version 1.3.1, ratios of retained introns to spliced introns (IR ratios) were computed[16]. Only transcripts tagged with either "-" or "MinorIsoform" on the warning column were chosen from those with adequate RNA expression (TPM value > 1). The highest IR ratio seen for any of the introns that were within a gene was chosen as the gene's representative IR ratio.

Classification of NMD sensitive genes

The list of NMD target transcripts was obtained from GENCODE annotation. The genes with sufficient expression ratios of NMD target transcripts (> 0.1 ratio) are

classified as NMD-sensitive. RNA isoform abundances were determined using quant of Kallisto version 0.44.0 with the default options[17]. Kallisto was performed on paired-end reads of RNA-Seq data to optimize the measurement of transcript abundances.

Comparison between the level of RPF mapped to RNA contaminants

Using the same alignment strategy applied to the genome, RPF was individually mapped to the rRNA, tRNA, and 7SL transcripts. If multiple primary alignments were present in the tRNA alignment, a single alignment was selected at random to solve the multiple alignment issue brought on by the homologous sequence between tRNA transcripts. For the alignment to 7SL, only the RPF mapped to the area encoding the S-domain was chosen in order to avoid any potential alignment issues brought on by the presence of multiple Alu elements inside the Alu-domain. The proportion of each RNA type in the infected samples was normalized by that in the uninfected controls.

Prediction of the ribosome stalling site and TMD selection

The site of ribosome stalling was estimated using the negative binomial probability distribution for ribosomal A-site coverage on the CDS region per transcript. Only the transcripts with adequate CDS length (>100 nt) and stalling sites with sufficient supporting reads (>10 reads) were taken into consideration in order to prevent potential bias from CDS length and an insufficient RPF expression level. A one-tailed negative binomial test was run for each stalling location to determine its significance. The true stalling sites were designated as the peak sites that are within the top 100,000 highest RPF density z-scores. For comparison between transcripts, the transcripts with at least one stalling site were defined as "stalled transcripts". The UniProtKB database was used to find the region of transmembrane domains in the transcripts that code for membrane proteins.

Identification of ribosome heterogeneity

DripARF was used to predict probable alterations in ribosomal protein abundance based on the position-specific RPF read count on the rRNA genome[18]. Positional rRNA abundance was measured before utilizing dripARF using genomecov of bedtools with the "-split" option. The heterogeneity contribution was evaluated by the normalized enrichment score (NES) of the Ribo-Seq reads that were aligned to RP contact sites of rRNAs. The human 80S ribosome crystal structure was retrieved from PDB (4UG0) for the ribosome structure plot and visualized with PyMOL version 2.5.4 (http://www.pymol.org/) utilizing a subset of rRNA and RP backbone residues[19].

Function and pathway enrichment analysis of translatomes and transcriptomes

Up- or down-regulated genes with the highest 10% or lowest 10% $\log_2$ fold change of expression are selected for each Ribo-Seq and RNA-Seq library from mouse lung

tissues or human cell line Calu-3[5]. Then, they are imported to the QIAGEN IPA (QIAGEN Inc., https://digitalinsights.qiagen.com/IPA)[20] software to perform function and pathway enrichment analysis in order to understand which biological functions are activated or depressed upon SARS-CoV-2 infection. To examine the difference in biological functions between up- or down-regulated genes from Ribo-Seq of mouse tissue and human cell line, a comparison analysis was carried out to analyze their results about diseases and functions. Terms are manually selected from comparison analysis and visualized. To find biological functions possibly regulated at the translation level, we assessed whether there are distinctions between expected biological changes for Ribo-Seq and RNA-Seq. After comparison analysis is performed using the results about diseases and functions, terms are selected manually among the statistically significant terms (B-H p-value < 0.01) to exclude terms that have duplicated biological meanings.

Comparison between up- or down-regulated genes identified from Ribo-Seq using mouse tissue and human cell line

The up- or down-regulated genes selected as we have said above are compared through the heatmap and Venn diagrams. Venn diagrams are drawn using ggVennDiagram package[21] in R v4.2.1. For the comparison, mouse-human homologues are acquired using biomaRt v2.48.3[22,23].

Reanalysis of the human COVID-19 single-cell transcriptome

A publicly available dataset of single-cell RNA-Seq (GSE171524)[24] is downloaded from Gene Expression Omnibus (GEO). Among the libraries, ones constructed with samples from donors who had acute pathogenesis (5–16 days from symptoms to death; L01cov, L03cov, L04cov, L08cov, and L09cov) are selected for comparison with the acute condition of mouse tissues. One control sample (C56ctr) was excluded because it formed an individual cluster in the initial Uniform Manifold Approximation and Projection (UMAP) analysis and showed heterogeneous characteristics. Data were processed using Seurat v4.1.1[25] in R v4.2.1. Cells that have unique feature counts less than 200 or over 3,000, and cells with over 4% mitochondrial counts, are filtered out. Then, data is normalized using the "NormalizeData" function with the "LogNormalize" method and 10,000 as a scale factor. 4,000 most variable genes were identified using the "FindVariableFeatures" function with the "vst" method. After scaling the data by applying the "ScaleData" function, principal component analysis (PCA) was performed with the variable genes. Next, UMAP was performed using 1–62 principal components (PCs) among 100 PCs. For the UMAP, the number of neighbors and minimum distance were set to 100 and 0.3, respectively. Differentially expressed genes (also called markers) of each cluster were identified by the "FindAllMarkers" function with the following criteria: only positive markers, at least 25% fraction, and 0.5 as the log fold change of threshold. The Human Protein Atlas[26] is used to manually label the cell types in clusters based on the markers that have been found.

The expression levels of gene sets are calculated by the “AddModuleScore” function using the single-cell expression levels of the up- or down-regulated genes selected. For the mouse genes, human homologues are acquired and applied for calculation.

To find out which genes are more expressed in COVID-19 or non-COVID-19 patients' lung cells, we used the "FindMarkers" function to compare cells from COVID-19 and non-COVID-19 samples for each cell type with the following options: only positive markers and 1 as the log fold change of threshold.

Statistical analysis

Ribosome stalling sites were determined using a one-tailed negative binomial test. For peak-calling analysis, an FDR cutoff of 0.05 was applied. As for GSEA, cellular component terms and KEGG terms with an adjusted P-value below 0.05 were considered as significant. FDR was calculated using the Benjamini-Hochberg method, and Cohen's d test was employed to compare the proportion of wobble C among specific GO terms and background genes. Intron retention ratios were calculated using IRFinder version 1.3.1, and genes with sufficient expression ratios of NMD target transcripts (>0.1 ratio) were classified as NMD-sensitive. As for function and pathway enrichment analysis, QIAGEN IPA software was utilized, and terms with B-H p-values less than 0.01 were considered significant.

Supplementary Method References

1. Martin, M. Cutadapt removes adapter sequences from high-throughput sequencing reads. *EMBnet.journal* **17**, 10-12 (2011).
2. Frankish, A. *et al.* Gencode 2021. *Nucleic Acids Res* **49**, D916-D923 (2021).
3. Kim, D., Paggi, J.M., Park, C., Bennett, C. & Salzberg, S.L. Graph-based genome alignment and genotyping with HISAT2 and HISAT-genotype. *Nat Biotechnol* **37**, 907-915 (2019).
4. Quinlan, A.R. & Hall, I.M. BEDTools: a flexible suite of utilities for comparing genomic features. *Bioinformatics* **26**, 841-842 (2010).
5. Finkel, Y. *et al.* SARS-CoV-2 uses a multipronged strategy to impede host protein synthesis. *Nature* **594**, 240-245 (2021).
6. Kim, D. *et al.* A high-resolution temporal atlas of the SARS-CoV-2 translatome and transcriptome. *Nat Commun* **12**, 5120 (2021).
7. Kim, D. *et al.* The Architecture of SARS-CoV-2 Transcriptome. *Cell* **181**, 914-921 e910 (2020).
8. Ahmed, N. *et al.* Identifying A- and P-site locations on ribosome-protected mRNA fragments using Integer Programming. *Sci Rep* **9**, 6256 (2019).
9. Uren, P.J. *et al.* Site identification in high-throughput RNA-protein interaction data. *Bioinformatics* **28**, 3013-3020 (2012).
10. Pedregosa, F. *et al.* Scikit-learn: Machine Learning in Python. *J Mach Learn Res* **12**, 2825-2830 (2011).
11. Bateman, A. *et al.* UniProt: the universal protein knowledgebase in 2021. *Nucleic Acids Res* **49**, D480-D489 (2021).
12. Subramanian, A. *et al.* Gene set enrichment analysis: a knowledge-based approach for interpreting genome-wide expression profiles. *Proc Natl Acad Sci U S A* **102**, 15545-15550 (2005).
13. Liberzon, A. *et al.* Molecular signatures database (MSigDB) 3.0. *Bioinformatics* **27**, 1739-1740 (2011).
14. Shannon, P. *et al.* Cytoscape: A software environment for integrated models of biomolecular interaction networks. *Genome Res* **13**, 2498-2504 (2003).
15. Bindea, G. *et al.* ClueGO: a Cytoscape plug-in to decipher functionally grouped gene ontology and pathway annotation networks. *Bioinformatics* **25**, 1091-1093 (2009).
16. Middleton, R. *et al.* IRFinder: assessing the impact of intron retention on mammalian gene expression. *Genome Biol* **18**(2017).
17. Bray, N.L., Pimentel, H., Melsted, P. & Pachter, L. Near-optimal probabilistic RNA-seq quantification. *Nat Biotechnol* **34**, 525-527 (2016).
18. Alkan, F. *et al.* Identifying ribosome heterogeneity using ribosome profiling. *Nucleic Acids Res* (2022).
19. Khatter, H., Myasnikov, A.G., Natchiar, S.K. & Klaholz, B.P. Structure of the human 80S ribosome. *Nature* **520**, 640-645 (2015).
20. Kramer, A., Green, J., Pollard, J., Jr. & Tugendreich, S. Causal analysis approaches in Ingenuity Pathway Analysis. *Bioinformatics* **30**, 523-530 (2014).

21. Gao, C.H., Yu, G. & Cai, P. ggVennDiagram: An Intuitive, Easy-to-Use, and Highly Customizable R Package to Generate Venn Diagram. *Front Genet* **12**, 706907 (2021).
22. Durinck, S. *et al.* BioMart and Bioconductor: a powerful link between biological databases and microarray data analysis. *Bioinformatics* **21**, 3439-3440 (2005).
23. Durinck, S., Spellman, P.T., Birney, E. & Huber, W. Mapping identifiers for the integration of genomic datasets with the R/Bioconductor package biomaRt. *Nat Protoc* **4**, 1184-1191 (2009).
24. Melms, J.C. *et al.* A molecular single-cell lung atlas of lethal COVID-19. *Nature* **595**, 114-119 (2021).
25. Hao, Y.H. *et al.* Integrated analysis of multimodal single-cell data. *Cell* **184**, 3573-+ (2021).
26. Thul, P.J. *et al.* A subcellular map of the human proteome. *Science* **356**(2017).

Supplementary Tables Brief Description

Supplementary Table 1. Three RPF abnormality indices (FrmI, UTRI, RlenI) of host and viral genes for each sample group. "FrmI" is the ratio of reads aligned to reading frame 1 of CDS. "UTRI" is the proportion of reads aligned to 3′ UTR versus CDS. "RlenI" is the percentage of short reads (< 30 nt) within the CDS. Each number corresponds to the average values of sample replicates.

Supplementary Table 2. Fold changes of mRNA, RPF, and RAI level of host nuclear genes. "FC of RNA level" and "FC of RPF level" columns present the fold change of RPM, and "FC of RAI level" present the difference between UTRI in the infected samples and the control.

Supplementary Table 3. Counts of canonical Ribo-seq and RNA-seq reads mapped to CDS regions of host nuclear genes. "RNA_count" and "RPF_count" sheets present the numbers of RNA-seq reads (only forward complement), and The canonical Ribo-seq reads (30–34 nt) mapped to CDS regions of individual genes.

Supplementary Table 4. Function and pathway terms overrepresented by IPA (Ingenuity Pathway Analysis) analysis with RPF and RNA level changes of individual genes, related to Fig. 6b. Enrichment scores (IPA Z scores), FDRs, and p-values of the function and pathway terms are included.

Supplementary Table 5. Information for the functionally grouped GO network in Fig. 6d. "Nodes_information" includes GSEA results of GO terms constituting the network. "Associated genes" indicate genes included in both the GO term and the list of genes used for enrichment. "Edges_information" shows Cohen's kappa coefficient values between GO terms.

Supplementary Table 6. Information on the GO enrichment network in Fig. 6e. "Nodes_information" includes GSEA results of GO terms constituting the network. "NES" contains normalised enrichment scores. "pV" indicates p-values in GSEA. "GS" means the number of genes associated with the enrichment of each term. "Edges_information" presents Sørensen–Dice coefficient between GO terms.

Supplementary Table 7. The list of non-COVID-19 and COVID-19 signature genes of each cell type cluster. "Mouse gene symbol" and "Mouse Ensembl gene ID" shows used homologues of signatures for the analysis.
